# Paradoxical clade- and age-specific vaccine effectiveness during the 2018/19 influenza A(H3N2) epidemic in Canada: potential imprint-regulated effect of vaccine (I-REV)

**DOI:** 10.2807/1560-7917.ES.2019.24.46.1900585

**Published:** 2019-11-14

**Authors:** Danuta M Skowronski, Suzana Sabaiduc, Siobhan Leir, Caren Rose, Macy Zou, Michelle Murti, James A Dickinson, Romy Olsha, Jonathan B Gubbay, Matthew A Croxen, Hugues Charest, Nathalie Bastien, Yan Li, Agatha Jassem, Mel Krajden, Gaston De Serres

**Affiliations:** 1 British Columbia Centre for Disease Control, Vancouver, Canada; 2 University of British Columbia, Vancouver, Canada; 3 Public Health Ontario, Toronto, Canada; 4 University of Toronto, Toronto, Canada; 5 University of Calgary, Calgary, Canada; 6 Alberta Precision Laboratories, Edmonton, Alberta; 7 University of Alberta, Edmonton, Canada; 8 Institut National de Santé Publique du Québec, Québec, Canada; 9 National Microbiology Laboratory, Public Health Agency of Canada, Winnipeg, Canada; 10 Laval University, Quebec, Canada; 11 Centre Hospitalier Universitaire de Québec, Québec, Canada

**Keywords:** Influenza, A(H3N2), imprinting, cohort effect, vaccine effectiveness, clade

## Abstract

**Introduction:**

The Canadian Sentinel Practitioner Surveillance Network reports vaccine effectiveness (VE) for the 2018/19 influenza A(H3N2) epidemic.

**Aim:**

To explain a paradoxical signal of increased clade 3C.3a risk among 35–54-year-old vaccinees, we hypothesise childhood immunological imprinting and a cohort effect following the 1968 influenza A(H3N2) pandemic.

**Methods:**

We assessed VE by test-negative design for influenza A(H3N2) overall and for co-circulating clades 3C.2a1b and 3C.3a. VE variation by age in 2018/19 was compared with amino acid variation in the haemagglutinin glycoprotein by year since 1968.

**Results:**

Influenza A(H3N2) VE was 17% (95% CI: −13 to 39) overall: 27% (95% CI: −7 to 50) for 3C.2a1b and −32% (95% CI: −119 to 21) for 3C.3a. Among 20–64-year-olds, VE was −7% (95% CI: −56 to 26): 6% (95% CI: −49 to 41) for 3C.2a1b and −96% (95% CI: −277 to −2) for 3C.3a. Clade 3C.3a VE showed a pronounced negative dip among 35–54-year-olds in whom the odds of medically attended illness were > 4-fold increased for vaccinated vs unvaccinated participants (p < 0.005). This age group was primed in childhood to influenza A(H3N2) viruses that for two decades following the 1968 pandemic bore a serine at haemagglutinin position 159, in common with contemporary 3C.3a viruses but mismatched to 3C.2a vaccine strains instead bearing tyrosine.

**Discussion:**

Imprinting by the first childhood influenza infection is known to confer long-lasting immunity focused toward priming epitopes. Our findings suggest vaccine mismatch may negatively interact with imprinted immunity. The immunological mechanisms for imprint-regulated effect of vaccine (I-REV) warrant investigation.

## Introduction

In the interim analysis for the 2018/19 influenza season, the Canadian Sentinel Practitioner Surveillance Network (SPSN) reported substantial vaccine effectiveness (VE) of 72% (95% confidence interval (CI): 60 to 81) for the primary influenza A(H1N1)pdm09 epidemic that peaked in late December and early January [1]. The SPSN also reported a prominent shift in the age distribution of influenza A(H1N1)pdm09 cases to include more children younger than 10 years. It was postulated that this reflected an immunological cohort effect following the 2009 influenza A(H1N1)pdm09 pandemic [2].

Thereafter, Canada experienced a secondary influenza A wave due to H3N2 subtype viruses that peaked unusually late, in March 2019 [3]. Several influenza A(H3N2) genetic clades contributed, the majority (> 60%) overall belonging to clade 3C.2a1b in Canada, Europe and Asia, with a minority (one-third or less) belonging to clade 3C.3a (Supplementary Figure S1) [3-7]. Conversely, clade 3C.3a viruses comprised the majority (> 70%) of influenza A(H3N2) detections in the United States (US) (Supplementary Figure S1) [7-10]. Clade 3C.3a viruses, which first arose in 2013/14, bear two substitutions within the immuno-dominant antigenic site B of the haemagglutinin (HA) glycoprotein. Both substitutions affect major antigenic cluster transition positions: one is clade-defining, namely a phenylalanine (F) to serine (S) substitution at HA position 159 (F159S), and another more recently acquired in 2016/17 is considered an accessory substitution, namely F193S [5-7,11-13].

The northern hemisphere influenza vaccine included a clade 3C.3a-representative virus in 2015/16, but clade 3C.3a viruses have since diversified [4-7]. In 2016/17, the influenza A(H3N2) vaccine component was updated to a clade 3C.2a strain, retained for 2017/18 but further updated in 2018/19 to clade 3C.2a1 [4]. With adaptation for egg-based manufacturing, clade 3C.2a vaccine candidates (including clade 3C.2a1) acquire several antigenically relevant mutations including a threonine (T) to lysine (K) substitution at HA position 160 of site B that constitutes an important loss of glycosylation (T160K (B) (−CHO)), exposing adjacent residues [14-17]. Antigenic characterisation by haemagglutination inhibition (HI) assay is problematic for all clade 3C.2a (and descendant viruses) [18]. Overall, however, egg-adapted 3C.2a and 3C.2a1 vaccine strains are considered antigenically related to each other but antigenically distinct from circulating 3C.2a1b and 3C.3a viruses, with greater antigenic distances generally recorded for 3C.3a [4-6].

Here, we report virological and VE findings for the 2018/19 influenza A(H3N2) epidemic in Canada. To explain a paradoxical signal of increased (rather than reduced) risk of clade 3C.3a illness among vaccinated compared with unvaccinated adults, we hypothesise an underlying cohort effect following the 1968 influenza A(H3N2) pandemic. The first influenza-priming infection in childhood is known to imprint the immune system, leading to long-lived memory responses that are focused towards pivotal epitopes of the imprinting virus [19,20]. We hypothesise that distant childhood priming provided long-lasting protection against contemporary influenza A(H3N2) viruses sharing imprinted epitopes, whereas vaccine mismatch may have negatively interacted with imprinted immunity. To explore the hypothesis of imprint-regulated effect of vaccine (I-REV), we compare VE variation by age in 2018/19 to amino acid variation in the HA glycoprotein by year since 1968.

## Methods

### Vaccine effectiveness estimation

Annual SPSN study activities are integrated within an outpatient sentinel surveillance platform for influenza-like illness (ILI) in the four most populous provinces of Canada. The ILI case definition is standardised for clinical severity, requiring both fever and cough plus additional systemic or respiratory symptoms [21]. The VE against medically attended, laboratory-confirmed ILI was estimated by test-negative design whereby the odds of vaccination in influenza test-positive cases were compared with influenza test-negative controls via the odds ratio (OR) [1,21]. The VE to the level of the influenza A(H3N2) genetic clade was derived as (1 − adjusted OR) × 100%, adjusted for potential confounders of age, province, specimen collection interval and calendar time. Comorbidity, sex and epidemic period were further explored in sensitivity analyses and Firth’s method of penalised logistic regression (PLR) addressed sparse data issues as specified [22-24]. The 2018/19 VE methods, influenza vaccine components and products (all egg-based) used in SPSN provinces are provided in Supplement S2.

### SPSN virus detection and characterisation

Specimens were tested for influenza by real-time RT-PCR. The genetic clade was determined by Sanger sequencing of the HA gene and phylogenetic analysis, with reference sequences obtained from the Global Initiative on Sharing All Influenza Data (GISAID) [25]. Amino acid substitutions at pivotal HA positions (H3 numbering with the signal peptide removed) are reported with antigenic sites indicated in parentheses (A–E), the receptor-binding site as ‘RBS’ and changes associated with potential gain or loss of N-linked glycosylation as ‘+/−CHO’. HA amino acid positions associated with major antigenic cluster transitions are specified [11-13,15,16]. Laboratory methods are provided in Supplement S3.

A convenience sample of virus isolates was also characterised antigenically by HI assay. Following regrowth of viruses in SIAT1 cells, the HI assay was conducted in the presence of 20 nM oseltamivir using guinea pig erythrocytes and post-infection ferret antisera raised against the 2018/19 egg-passaged vaccine reference virus [26].

### Identification of historic priming (imprinting) epochs

All available HA sequences from influenza A(H3N2) viruses with collection dates from 1 January 1968 to 31 July 2019 were downloaded from GISAID [25] and aligned using MAFFT v7.245 [27]. We gratefully acknowledge the hundreds of authors, originating and submitting laboratories that contributed sequences to GISAID since 1968, enabling the phylogenetic and priming epoch analyses we report here.

Amino acids at select antigenic site positions were extracted using python scripts and their distribution compared by 1-year and 5-year groupings across historic and contemporary influenza A(H3N2) viruses, including the 2018/19 egg-adapted 3C.2a1 vaccine and wild-type 3C.2a1b and 3C.3a consensus sequences. Corresponding birth years were derived as 2018 minus age. Selected antigenic site positions were associated with clade-defining or other antigenic cluster transition or accessory substitutions or with potential gain or loss of glycosylation.

Individuals 55 years and older in 2018/19 would probably have been primed to A(H2N2) (emergent in 1957) or A(H1N1) (emergent in 1918) viruses and children aged 10–19 years may have been primed to influenza A(H1N1)pdm09. These subtypes belong to group 1 HA, whereas A(H3N2) viruses belong to group 2 HA [19]. Children younger than 5 years may not yet have had an influenza priming exposure [19,28].

Cohort effects require highly prevalent and/or persistent epitope alterations (e.g. following pandemics or major antigenic cluster transitions) to be detectible at the population level. In that context, minor variations or data inconsistencies were less critical to address and further cleaning of downloaded GISAID data was not undertaken. 

### Clade- and age-specific vaccine effectiveness: exploratory cohort (imprinting) effects

Adult age subsets were regrouped based on potential priming epochs, taking into account a possible 4–5-year delay from birth to first childhood influenza infection [19,28]. Case distributions and VE estimates, stratified by influenza A(H3N2) clade, were assessed by these redefined age subsets. The VE by single year of age was also explored among participants 1–64 years-old (owing to sparse data in elderly adults 65 years and older) with age smoothed as a natural cubic spline function (for detailed methods see Supplement S2).

### Ethical statement

The 2018/19 VE study protocol was approved by ethics review committees: University of Calgary, Calgary, Alberta (REB15–0587_MOD8); University of British Columbia, Vancouver, British Columbia (H04–80634); Public Health Ontario, Toronto, Ontario (2017–057.02); and Comité d’éthique de santé publique, Québec.

## Results

Between 1 November 2018 and 30 April 2019, 1,993 specimens were eligible for estimation of VE against influenza A(H3N2), including 332 cases (peaking in March 2019) and 1,661 controls (Supplementary Figure S4).

### Influenza A(H3N2) virological characterisation

The HA gene of 96% (318/332) of contributing A(H3N2) viruses were sequenced (GISAID numbers for 315 of them: EPI_ISL_346183–346213 and EPI_ISL_394581–394864). Clade distribution is shown in Supplementary Table S5, displayed by phylogenetic clusters in Supplementary Figure S6 and by week in Supplementary Figure S7.

Overall, 58% (184/318) of A(H3N2) viruses belonged to clade 3C.2a1b, of which 79% (145/184) bore an extra T131K(A) substitution and 21% (39/184) instead bore an extra T135K(A)(RBS)(−CHO) substitution; 35% (110/318) belonged to clade 3C.3a.

We attempted HI characterisation on one-third (109/332) of A(H3N2) viruses, successful for 11 of 54 clade 3C.2a1b and 49 of 50 clade 3C.3a viruses. Of the viruses successfully HI-characterised, four of the 11 3C.2a1b (3/4 with T135K and 1/7 with T131K) and 38 of the 49 3C.3a viruses were antigenically distinct from the vaccine strain.

### Participant profiles

As in prior SPSN analyses, most (> 60%) participants were age 20–64 years, and ca one-third were vaccinated [21]. For detailed profiles, see Supplement S8.

### Clade- and age-specific vaccine effectiveness: primary analyses

**Figure 1:**
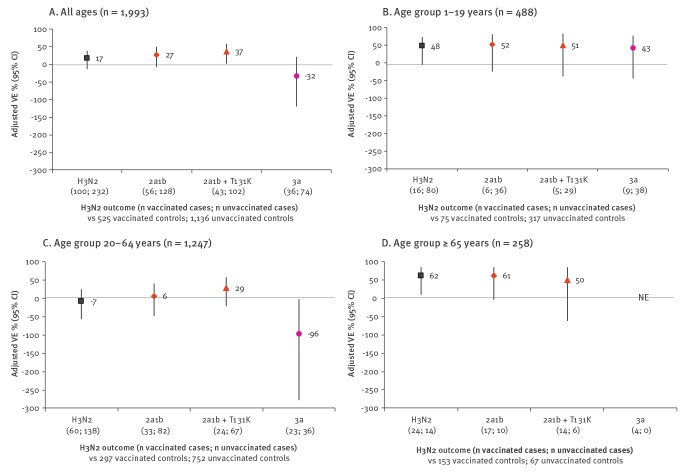
Vaccine effectiveness against influenza A(H3N2) viruses overall and by age and genetic subgroup, Canadian Sentinel Practitioner Surveillance Network, 2018/19 (n = 1,993)

CI: confidence interval; NE: not estimated; VE: vaccine effectiveness.

Influenza A(H3N2) VE was 17% (95% CI: −13 to 39) overall: 27% (95% CI: −7 to 50) for 3C.2a1b and −32% (95% CI: −119 to 21) for 3C.3a (Figure 1). VE was 48% (95% CI: −5 to 74) for children 1–19 years-old but −7% (95% CI: −56 to 26) for adults 20–64 years-old, the latter reflecting a significant negative VE against clade 3C.3a (−96%; 95% CI: −277 to −2) but not 3C.2a1b (6%; 95% CI: −49 to 41). The findings did not meaningfully differ in sensitivity analyses (Supplementary Table S9).

Adjusted VE estimates with 95% CI are displayed. All VE estimates are adjusted for age group, province, specimen collection interval and calendar time. Methods are detailed in Supplement S2 and model specifications and VE estimates with precise CI are provided in Supplementary Table S9.

Among participants 9 years and older, 87% of influenza A(H3N2) cases and 94% of controls who reported 2018/19 vaccination also reported 2017/18 vaccination, with lower VE among repeat recipients (−1%; 95% CI: −45 to 29) compared with those who received the vaccine in 2018/19 only (69%; 95% CI: 18 to 89) (Supplementary Table S10). Of the 30 clade 3C.3a cases that were 9 years and older and vaccinated in 2018/19, all reported also being vaccinated in 2017/18; VE against clade 3C.3a for dually vaccinated relative to dually unvaccinated participants was −115% (95% CI: −304 to −15).

### Age distribution of cases and controls

During previous influenza A(H3N2) epidemics in 2014/15, 2016/17 and 2017/18, the percentage of unvaccinated influenza A(H3N2) cases by single year of age was evenly distributed across the age range. In these three seasons, the median age of influenza A(H3N2) cases was consistently at least 30 years (33, 30 and 36.5 years, respectively) and did not differ significantly from controls (30, 31 and 34 years, respectively) [2]. Vaccinated cases were older, with median age consistently more than 50 years (53, 54 and 57 years, respectively), which was also not significantly different from controls (52, 55 and 52 years, respectively) (Supplementary Figure S11).

**Figure 2:**
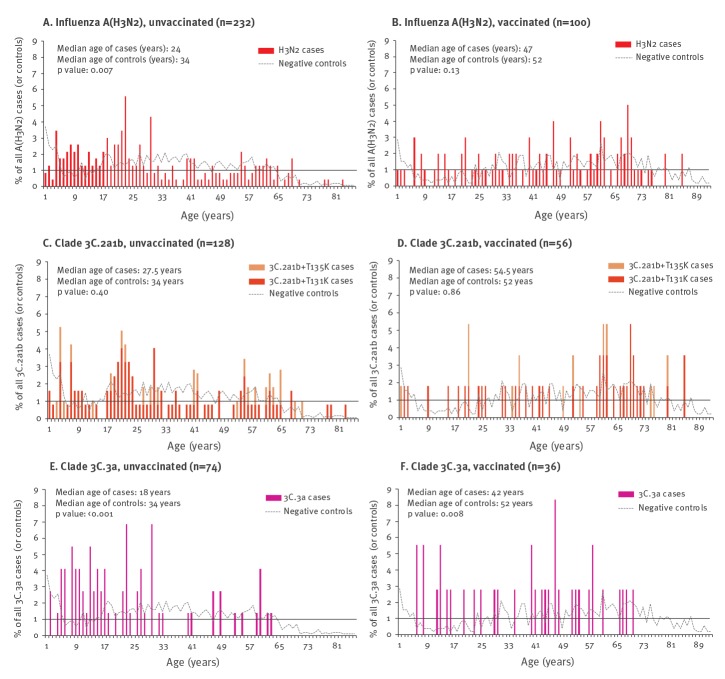
Percentage histogram of influenza A(H3N2) cases (overall and clade-specific) and controls by single year of age and vaccination status, Canadian Sentinel Practitioner Surveillance Network, 2018/19 (n = 1,993)

CI: confidence interval; VE: vaccine effectiveness.

In 2018/19, however, more unvaccinated influenza A(H3N2) cases were younger than 30 years, with significantly younger median age compared with unvaccinated controls (24 vs 34 years; p = 0.007) (Figure 2). By clade, unvaccinated 3C.3a cases were significantly younger than 3C.2a1b cases (18 vs 27.5 years, respectively; p = 0.003), with median age of 3C.3a cases (p < 0.001) but not 3C.2a1b cases (p = 0.40) significantly different from controls. Vaccinated influenza A(H3N2) cases were also younger than vaccinated controls (47 vs 52 years; p = 0.13), with 3C.3a cases younger than 3C.2a1b cases (42 years vs 54.5 years; p = 0.01) and median age of 3C.3a cases (p = 0.008) but not 3C.2a1b cases (p = 0.86) significantly younger than controls.

Age distributions are displayed for unvaccinated (left side) and vaccinated (right side) study participants for A(H3N2) cases overall (panels A and B), for clade 3C.2a1b (further stratified by cases with T135K or T131K substitution) (panels C and D) and for clade 3C.3a (panels E and F). The percentages of all influenza A(H3N2) cases belonging to a given single year of age are displayed as coloured bars. The same information for influenza test-negative controls is superimposed as a dotted line to indicate the sampling distribution; note that the control distribution is the same in each panel. The p values displayed are for the comparison between median ages of cases and controls within the same season. Vaccination status is based on self-report (or by parent/guardian) of receiving at least one dose of influenza vaccine at least 2 weeks before onset of influenza-like illness.

### Potential priming epochs

To explain a paucity of unvaccinated 3C.3a cases older than 30 years, we sought priming epitopes to which adults may have been uniquely exposed in childhood and which were shared with contemporary 3C.3a viruses but not with 3C.2a1 vaccine and circulating 3C.2a1b viruses. We assessed amino-acid variation at 32 HA antigenic site positions across 83,026 historic influenza A(H3) sequences since 1968, aligned with 5-year age intervals in 2018/19 and corresponding birth years (Supplementary Table S12). We propose that HA position 159 played a central role with potential accessory contribution by position 193.

**Figure 3:**
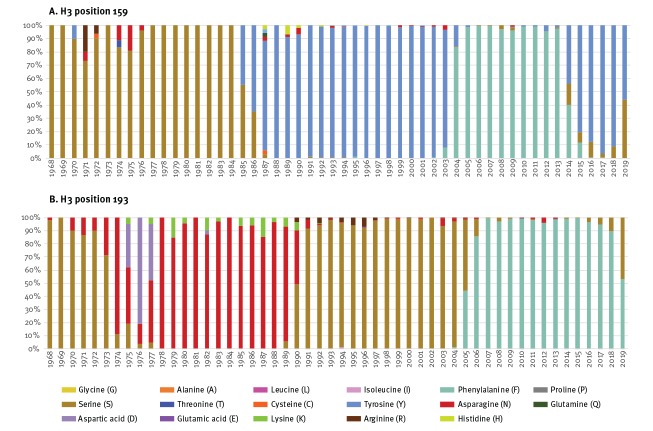
Percentage of worldwide influenza A(H3N2) viruses with specified amino acid residues at haemagglutinin (H3) positions 159 and 193, by year, GISAID, 1968–2019 (n = 83,026)

As shown in Figure 3, A(H3N2) viruses bore serine at position 159 (S159, like clade 3C.3a) for nearly two decades following the 1968 pandemic, but have not possessed S159 since the emergence of A/Sichuan/2/1987-like viruses ca 30 years ago. In regard to position 193, A(H3N2) viruses last bore S193 (like 3C.3a viruses) ca 15 years ago and also bore S193 briefly from 1968 until ca 1973.

GISAID: Global Initiative on Sharing All Influenza Data.

The proportion of influenza A(H3N2) viruses with the specified amino acids at positions 159 (panel A) and 193 (panel B) are displayed by year since 1968; actual tallies by year are provided in Supplementary Table S13. Note that the number of A(H3) sequences is not consistent from year to year, with generally less than 100 A(H3) sequences per year until the 1990s (excepting 1968), less than 1,000 per year until 2003 and more than 1,000 per year thereafter available for download from GISAID.

Conversely, contemporary 3C.2a and descendant viruses, including 3C.2a1 vaccine and 3C.2a1b circulating viruses, possess Y159 and F193 (Supplementary Table S12). Of related importance, no A(H3N2) viruses since 1968 possessed the N158/T160 glycosylation motif that was newly acquired by 3C.2a viruses from 2013/14. With addition of that sugar moiety, position 159 in 3C.2a and descendant viruses (including 3C.2a1b) is shielded from antibody and other immune system access. During the egg adaptation process, however, 3C.2a and 3C.2a1 vaccine strains lose that glycosylation site and associated shielding of position 159.

### Age regrouping based on potential priming epochs

To reflect potential priming epochs, we redefined adult age subsets as 20–34, 35–54 and 55–64 years.

As shown in Supplementary Figure S14, children younger than 20 years were proportionately over-represented among both unvaccinated and vaccinated 3C.3a cases compared with 3C.2a1b cases or controls. Conversely, adults 35–54-years-old were under-represented among unvaccinated 3C.3a cases compared with unvaccinated 3C.2a1b cases or controls (7/74 (9%) vs 22/128 (17%) or 327/1,136 (29%)) but were over-represented among vaccinated counterparts (14/36 (39%) vs 12/56 (21%) or 140/525 (27%)). 

Overall, for adults 35–54-years-old, the odds of clade 3C.3a illness were 4.67 times greater (95% CI: 1.85 to 11.82) among vaccinated compared with unvaccinated participants (p < 0.001) (Supplementary Figure S15). In addition, the odds of being vaccinated rather than unvaccinated were 3.67 times greater (95% CI: 1.16 to 11.56) for 3C.3a than for 3C.2a1b cases (p = 0.02). There were no other significant differences in clade-specific risk by vaccination status for any other age group in this crude analysis.

### Clade- and age-specific vaccine effectiveness – exploratory cohort (imprinting) effects

**Figure 4:**
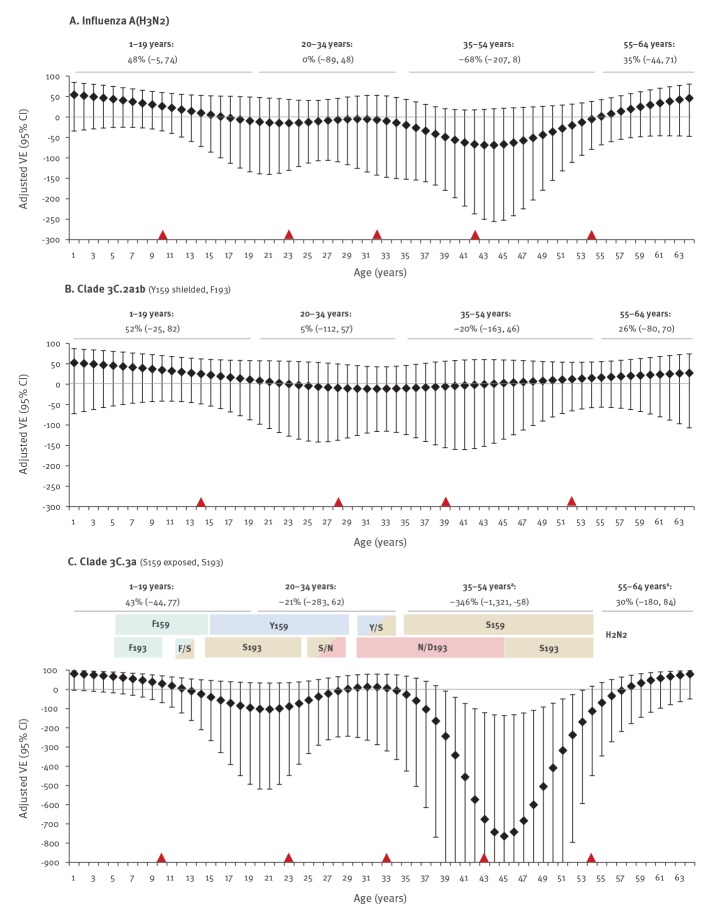
Overall and clade-specific vaccine effectiveness against influenza A(H3N2), explored by age modelled by single year, Canadian Sentinel Practitioner Surveillance Network, 2018/19 (n = 1,735)

CI: confidence interval; HA: haemagglutinin; VE: vaccine effectiveness.

To further explore potential cohort (imprinting) effects, we assessed VE by single year of age. Splines are shown in Figure 4 with VE estimates by age subset overlaid; colour shading in the 3C.3a panel reflects potential priming epochs associated with HA positions 159 and 193.

Adjusted VE estimates with 95% CI are displayed for A(H3N2) viruses overall (panel A) and for 3C.2a1b (panel B) and 3C.3a (panel C) viruses. Amino acids at HA positions 159 and 193 of 3C.2a1b and 3C.3a viruses are specified in panel labels, including whether position 159 was shielded or exposed (reflecting presence or absence of adjacent glycosylation); for comparison, note that the 2018/19 3C.2a1 vaccine is Y159-exposed, F193. VE estimates by single year of age are presented among participants aged 1–64 years with age smoothed as a natural cubic spline function assessed with knots at equal percentiles of age, demarcated by red triangles. Adjusted VE estimates by age subset are also overlaid. Methods are detailed in Supplement S2 and age subset model specifications and VE estimates with confidence intervals are provided in Supplement S9. For panel C related to 3C.3a, variation in amino acids at HA positions 159/193 of historic A(H3N2) viruses is overlaid with colour shading, corresponding to potential childhood priming epochs associated with those amino acid positions for participants of the specified age in 2018/19.

^a^ Firth’s method of penalised logistic regression was used owing to limited sample size.

These exploratory analyses show vaccine protection in children 1–19-years-old that declined towards the null with increasing age through childhood overall and for both clades 3C.2a1b and 3C.3a. Vaccine protection was also suggested among older adults 55–64 years.

In younger adults, however, age-related patterns differed by genetic clade. For 3C.2a1b viruses that were Y159/F193 matched to the vaccine but with position 159 shielded from antibody access, VE hovered around the null throughout adulthood with point estimates varying at most 20% above or below. Conversely, for 3C.3a viruses that were S159/S193 vaccine-mismatched and with position 159 unshielded, a pronounced drop in VE below the null was evident between ca 35 and 54 years of age. Negative VE for adults 35–54-years-old (−346%; 95% CI: −58 to −1,321, Firth's PLR) corresponds with a 4.46-fold increased risk (95% CI: 1.58 to 13.21) among vaccinated individuals (p < 0.005) (Figure 4; Supplementary Table S9). A lesser dip below the null was also observed among participants ca 13–28 years-old (VE of −57%; 95% CI: −349 to 48, Firth’s PLR), corresponding to birth cohorts likely to have been primed to influenza A(H3N2) viruses that were Y159-matched but S193-mismatched to the 2018/19 (Y159/F193) vaccine.

Negative VE in middle-aged adults was not identified for prior influenza A(H3N2) epidemics in 2016/17 or 2017/18 (Supplementary Figure S16). For the 2017/18 influenza A(H3N2) epidemic that was due mostly (> 85%) to 3C.2a2 (Y159/F193 vaccine-matched) viruses, significant positive VE (40%; 95% CI: 7 to 61) was instead observed for adults 35–54-years-old, whereas for all other ages, VE hovered around the null. This mirror-image pattern compared with 2018/19 may be a clue to possible underlying mechanisms.

### Assessment for bias

We adjusted for potential confounders of age, province, specimen collection interval and calendar time; in sensitivity analyses, further adjustment for comorbidity and sex and restriction by epidemic period did not meaningfully alter findings (Supplementary Table S9). We further scrutinised for signals of selection bias as per below.

### Review of participant profiles

National coverage surveys for 2018/19 indicate that among Canadians 18–64-years-old, 31% without comorbidity and 43% with comorbidity received influenza vaccine, as did 70% of elderly adults overall [3]. Similarly, among our test-negative controls, 27% (231/853) of those aged 18–64 years without comorbidity and 43% (98/226) with comorbidity self-reported any influenza vaccination (without reference to timing in relation to ILI onset), as did 30% (107/363) and 46% (44/95), respectively, of 35–54-year-olds and 72% (173/240) of elderly adults overall. In our VE analyses, the greater proportion of vaccinated 3C.3a cases was not explained by an excess with known (i.e. excluding unknown) comorbidities overall (15/99; 15%) or among adults 35–54 years (3/15; 20%) relative to controls overall (363/1,571; 23%) or among adults 35-54 years (88/443; 20%) or compared with other surveillance data indicating > 20% of Canadians live with a major chronic disease [29].

### Influenza A(H1N1)pdm09 vaccine effectiveness analysis

If selection bias were operating in our dataset it should also affect influenza A(H1N1)pdm09 estimates. However, end-of-season VE for influenza A(H1N1)pdm09 was substantial (67%; 95% CI: 58 to 75), comparable to mid-season [1] and significantly higher than for influenza A(H3N2) overall (17%; 95% CI: −13 to 39), including for influenza A(H1N1)pdm09 among participants 20–64 years-old (63%; 95% CI: 50 to 72) and 35–54 years-old (66%; 95% CI: 48 to 78).

### Sham vaccine effectiveness analysis

In the absence of selection bias, the OR for vaccine effect against non-influenza respiratory viruses should approximate 1.0 [30]. During the 2018/19 season, two provinces conducted routine testing for respiratory syncytial virus (RSV). Applying the same covariates, the OR for vaccine effect against RSV (131 cases and 955 controls) was 1.04 (95% CI: 0.66 to 1.62) and with additional adjustment for comorbidity and sex was 1.00 (95% CI: 0.63 to 1.58). With restriction to adults 35–54 years (30 cases, 277 controls) the OR for RSV was 1.25 (95% CI: 0.56 to 2.80). The latter does not approach the substantial OR for 3C.3a viruses in 35–54-year-olds in these RSV-testing provinces (3.75; 95% CI: 1.06 to 16.28, Firth’s PLR) (12 cases, 308 controls). Note that in an analysis by test-negative design, a negative VE for RSV would mean that including RSV cases as controls in the evaluation of influenza VE would tend to under-estimate a true negative vaccine effect for influenza.

## Discussion

During the 2018/19 late-season influenza A(H3N2) epidemic, the Canadian SPSN observed low VE against outpatient influenza A(H3N2) illness, including significant negative VE for clade 3C.3a viruses among adults 20–64-years-old (−96%), pronounced among adults 35–54-years-old (−346%). A similar signal of negative VE for clade 3C.3a illness was identified in 2018/19 by the European I-MOVE primary care network among adults 15–64 years (−74%; 95% CI: −259 to 6) [31]. In the US where clade 3C.3a viruses predominated, the Hospitalized Adult Influenza Vaccine Effectiveness Network (HAIVEN) reported negative VE against influenza A(H3N2) overall (−43%; 95% CI: −102 to −2), a finding also driven by non-elderly adults that remains under investigation [8]. The US outpatient FluVE network also reported negative (albeit non-significant) VE against clade 3C.3a illness among 18–49-year-olds (−10%; 95% CI: −47 to 18) and 50–64-year-olds (−48%; 95% CI: −142 to 10) [10]. These separate international networks differ somewhat in the methods, vaccines (e.g. egg- vs cell-based) and laboratory-confirmed clinical outcomes (e.g. outpatient vs inpatient; acute respiratory illness vs ILI) that were used. Although findings become less robust with stratification and reduced sample size, consistency in the direction of the negative vaccine effect against clade 3C.3a viruses among non-elderly adults across these networks requires explanation beyond chance variation.

In the absence of an obvious indication of bias, we have considered a biological phenomenon. Clade- and age-specific observations suggest an underlying cohort effect which we postulate may be linked to the distant but durable immunological imprint made by the first influenza virus exposure of childhood. This concept of imprinting was initially described by Davenport in 1957 with negative branding as *Original Antigenic Sin* by Francis in 1960 [32-34]. The 2009 influenza A(H1N1) pandemic demonstrated the potential protective effects of childhood imprinting against recycled epitopes in the very old, and since then, the phenomenon has received more recognition [35]. The potential influence of imprinting on age-related susceptibility across novel subtypes within the same HA groups (i.e. group 1 vs group 2) [36-39], or across genetic variants within the same subtype [40-44] has more recently been described. It has also been suggested that immunological history needs to be taken into account in influenza VE evaluation and interpretation [45-47].

We sought a unifying hypothesis to simultaneously explain signals of pre-existing clade 3C.3a protection in unvaccinated adults older than 30 years and increased risk among their vaccinated counterparts. Taking into account a potential delay of several years from birth to first influenza-priming exposure [19,28], we postulate that individuals 35–54-years-old in 2018/19, corresponding to birth years 1964 to 1983, acquired protection through imprinting to historic influenza A(H3N2) viruses that bore S159 in common with contemporary clade 3C.3a viruses. Position 159 of the HA head is a focal point for immune response [11] and S159 was an accessible trigger for immunological imprinting and memory back-boosting throughout that 20-year period.

Negative VE for clade 3C.3a viruses was driven by the majority (> 80%) of vaccinees in our dataset who were repeat recipients of Y159-bearing 3C.2a vaccines in 2017/18 and 2018/19, mismatched to contemporary S159-bearing clade 3C.3a as well as distant imprinting viruses. In that context, the antigenic-distance hypothesis (ADH) previously elaborated by Smith et al. predicts negative interference from the prior season’s vaccine, potentially reducing VE [48,49]; however, the ADH did not incorporate more distant immunological interactions nor did it allow for negative VE (vaccine-associated increased risk). In order for our I-REV hypothesis to explain not only reduced or null, but also negative VE, we suggest that mismatched epitope responses must have somehow interfered with imprinted immunity. In a recent ferret study involving influenza A(H1N1) viruses, the cross-reactivity of imprinted A/USSR/90/1977 antibody against antigenically distinct A/Taiwan/1/1986 was diminished with each additional dose of antigenically distinct A/California/07/2009 vaccine received [20]. Similar epitope narrowing of imprinted cross-protection with repeat vaccination may have contributed to our findings. Antibody-dependent enhancement (ADE) of viral replication may also explain vaccine-associated increased risk. ADE of influenza infection has long been postulated [50], including for heterologous vaccination in swine [51,52], but is not generally accepted. For other diseases, ADE is described during particular periods (windows of opportunity) when weakly cross-reactive antibodies are present at precise low levels [53,54], such as might variably be the conditions during late-season epidemics caused by antigenically distinct or drifted virus. The Canadian SPSN previously raised this possibility in relation to heterologous seasonal vaccination and increased risk of influenza A(H1N1)pdm09 illness during the spring wave of the 2009 pandemic [55], recapitulated in ferrets [56] and hypothesised by others to be mediated by immune-complex formation [57]. The immunological mechanisms underlying I-REV require specific investigation in ferret or other experimental models.

Ultimately, our findings constitute a strong surveillance signal with consistency across VE networks and a concordant hypothesis, but are based on observational design and sparse data. Given the multivalent vaccine, policy implications must take into account not only negative clade- and age-specific VE against influenza A(H3N2) illness but also the protective effects of other vaccine components (e.g. against H1N1pdm09 illness) for the same age group and season. It is important to underscore that as a weighted average of any influenza type/subtype contribution, vaccine was protective during the 2018/19 season in Canada with a VE of 56% (95% CI: 47 to 64) overall and 49% (95% CI: 28 to 64) for adults 35–54-years-old. Nevertheless, better understanding of variation in influenza VE is needed, incorporating distant imprinting as well as proximate influences and interactions, with a view to improving influenza vaccine design and performance over the long term. We offer the I-REV hypothesis (Box) as springboard for discussion by the broader scientific community, recognising that other viral components (e.g. HA stalk, neuraminidase) and mechanisms may play a role and that under some conditions, childhood immunological imprinting has beneficial effects.

Box **Imprint-regulated effect of vaccine (I-REV)**

**Supporting rationale:**

- The first (priming) influenza virus infection in childhood imprints the immune system, leading to long-lived memory responses focused towards pivotal epitopes of the imprinting virus.
- Most people have had a first influenza virus infection before the age of 4–5 years [19,28].
- Position 159 of the influenza A(H3) glycoprotein is a focal point for immune system interaction; position 193 may play an accessory role [11].
- Until about 30 years ago, for two decades following the 1968 pandemic, influenza A(H3N2) viruses bore S159; no glycosylation site shielded position 159 from the immune system during that period.
- Contemporary 3C.2a viruses^a^ possess Y159; however, position 159 is shielded from the immune system because of adjacent T160 glycosylation.
- Contemporary 3C.2a vaccine strains^b^ also possess Y159; however, position 159 is exposed to the immune system because of adjacent T160K loss of glycosylation following adaptation for egg-based manufacturing [14].
- Contemporary 3C.3a viruses possess S159; position 159 is also exposed to the immune system because clade 3C.3a viruses are naturally K160-non-glycosylated.

**I-REV hypothesis:**

- Distant childhood priming and memory back-boosting of S159 responses following the 1968 pandemic conferred long-lasting immunity to imprinted cohorts, protecting unvaccinated adults aged 35–54 years in 2018/19 (birth cohorts 1964–83) from clade 3C.3a viruses that also bore S159.
- Mismatched vaccination with egg-adapted 3C.2a antigen^b^ instead bearing Y159 may have negatively interacted with imprinted immunity^c^.
- The underlying immunological mechanisms for the I-REV hypothesis require further investigation.

^a^ Clade 3C.2a1b viruses in particular predominated during the 2018/19 influenza A(H3N2) epidemic.
^b^ In addition to the 2018/19 clade 3C.2a1 vaccine, the egg-adapted 2016/17 and 2017/18 clade 3C.2a vaccine strains were also non-glycosylated at Y159 (bearing T160K) and also bore F193. Repeat vaccination effects may have played a role in I-REV.
^c^ Although not unique to adults older than 30 years, S193 imprinting may have played an accessory role.

## Supplementary Data

1933000 Supplement_Skowronski_19-00585
