# Supplementary material for: Paradoxical clade- and age-specific vaccine effectiveness during the 2018/19 influenza A(H3N2) epidemic in Canada: potential imprint-regulated effect of vaccine (I-REV)
Source: Euro Surveill. 2019 Nov 14;24(46):1900585. doi: 10.2807/1560-7917.ES.2019.24.46.1900585 (PMC6864978; doi:10.2807/1560-7917.ES.2019.24.46.1900585)
Supplement: Supplement_Skowronski_19-00585 [file 19-00585_SKOWRONSKI_Supplement.pdf]

Supplementary Materials

This supplementary material is hosted by *Eurosurveillance* as supporting information alongside the article "*Paradoxical clade- and age-specific vaccine effectiveness during the 2018/19 influenza A(H3N2) epidemic: potential imprint-regulated effect of vaccine (I-REV)*" on behalf of the authors who remain responsible for the accuracy and appropriateness of the content. The same standards for ethics, copyright, attributions and permissions as for the article apply. Supplements are not edited by Eurosurveillance and the journal is not responsible for the maintenance of any links or email addresses provided therein.

Supplementary Material Contents (hyperlinked)

Supplement Figure S1. Influenza A(H3N2) genetic subgroup distribution: Canada, Europe, Asia, United States, 2018/19 3

Supplement S2. Influenza vaccine effectiveness (VE) methods and products 4

Table S2A. Northern hemisphere influenza vaccine components, 2017/18 and 2018/19 6

Table S2B. Products by percentage of publicly-funded doses distributed, SPSN provinces, 2018/19 6

Supplement S3. Laboratory methods 7

Table S3. Influenza screening and subtyping assays by province 8

Supplement Figure S4. Epidemic curve by influenza type/subtype and week, Canadian SPSN, 2018/19 9

Supplement Table S5. Summary of influenza A(H3N2) cases by genetic subgroup 10

Supplement Figure S6. Phylogenetic tree, influenza A(H3N2) viruses 11

Supplement Figure S7. Epidemic curve by influenza A(H3N2) genetic subgroup and week 12

Supplement S8. Participant characteristics Canadian SPSN, 2018/19 13

Table S8A. Overall influenza A(H3N2) cases and controls 14

Table S8B. Clade 3C.2a1b cases and controls 15

Table S8C. Clade 3C.3a cases and controls 16

Supplement Table S9. Influenza A(H3N2) vaccine effectiveness overall and by genetic subgroup, Canadian SPSN, 2018/19 17

Table S9 Cont'd. VE against predominant 3C.2a1b subgroup with T131K substitution 18

Supplement Table S10. Repeat vaccination effects: influenza A(H3N2) overall and by genetic clade 19

Table S10A. Stratified analysis - 4-level indicator variable 19

Table S10B. Stratified analysis - 2-level subset restriction 19

Supplement Figure S11. Percentage histogram of vaccinated influenza A(H3N2) cases and controls by single year of age, 2014/15, 2016/17, and 2017/18 epidemics 20

Supplement Table S12. Amino-acid variation at select antigenic sites of historic influenza A(H3N2) viruses since 1968, aligned by 5-year age bands and corresponding birth cohorts reflecting potential priming epochs 21

Supplement Table S13. Global influenza A(H3) position 159 and 193 amino acid tallies, by year, 1968 to 2019 23

Supplement Figure S14. Percentage histogram of clade 3C.2a1b and clade 3C.3a cases and controls by age subset and vaccination status 24

Supplement Figure S15. Percentage vaccinated among clade 3C.2a1b and clade 3C.3a cases and controls by age subset 25

Supplement Figure S16. Influenza A(H3N2) vaccine effectiveness by single year of age, 2016/17, 2017/18 and 2018/19 26

Supplement S17. References 27

Supplement Figure S1. Influenza A(H3N2) genetic subgroup distribution: Canada, Europe, Asia, United States, 2018/19

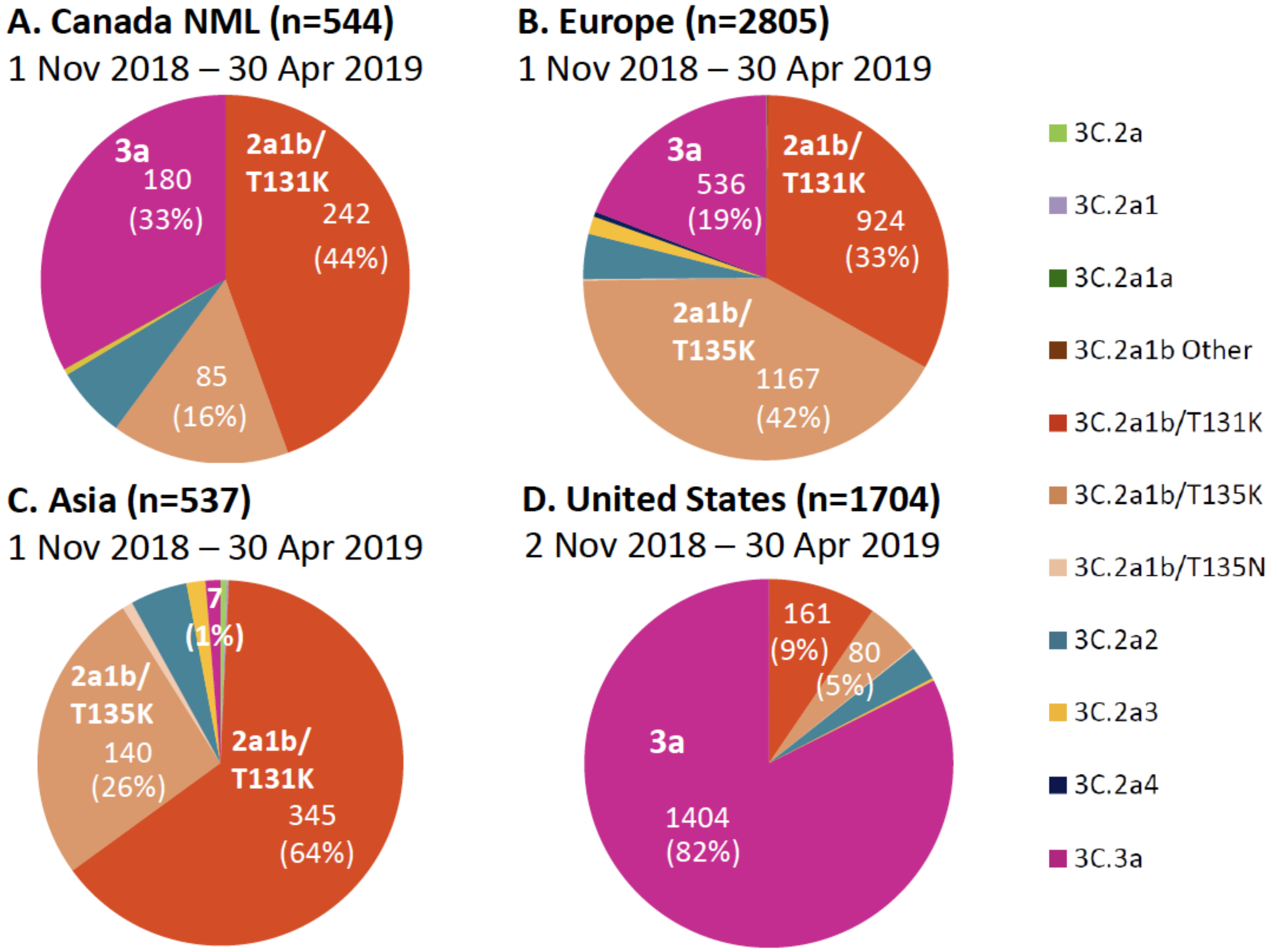

The 2018/19 hemagglutinin (HA) genetic subgroup distribution of influenza A(H3N2) viruses based on sequences uploaded to the Global Initiative on Sharing All Influenza Data (GISAID: www.gisaid.org) by Canada's National Microbiology Laboratory (NML) (panel A); as well as contributing laboratories from 38 countries of Europe (panel B); Asia (including Japan, Hong Kong SAR and China) (panel C); and for the United States (panel D). Sequences with collection dates spanning 1 November 2018 and 30 April 2019 are displayed, current as of download from GISAID on 22 August 2019 [1].

The actual tally (and proportion) of 3C.2a1b viruses with T131K or T135K and of 3C.3a viruses are displayed for each region; all other genetic subgroups comprised <10% each of all A(H3N2) sequences available for analysis for each of the regions displayed.

Note that variation in clade distribution may reflect the sampling framework of included viruses/sequences such as contributing jurisdictions, mix of inpatients/outpatients, age groups etc.

Supplement S2. Influenza vaccine effectiveness (VE) methods and products

Participant recruitment

The Canadian Sentinel Practitioner Surveillance Network (SPSN) includes sentinel outpatient sites in the four most populous provinces of Canada (together comprising >85% of the Canadian population) [2]: Alberta, British Columbia, Ontario and Quebec. Patients presenting to a sentinel site between 1 November and 30 April were eligible for inclusion in VE analysis if ≥1-year-old and attending within 7 days of onset of influenza-like illness (ILI), defined as self-reported fever and cough and at least one other symptom of sore throat, myalgia, arthralgia or prostration; fever is not required for elderly adults ≥65-years-old. Epidemiological information is collected by sentinel practitioners using a standard questionnaire at nasal/nasopharyngeal specimen collection before test result is known.

Influenza vaccine effectiveness (VE) analyses – primary and age subset

Vaccine components are shown below in Table S2A and vaccine products used in SPSN provinces in Table S2B. VE estimates were derived by test-negative design as described in prior publications by the SPSN [3,4]. Patients testing positive for influenza were cases; those testing negative for any influenza viruses were controls. Cases were assessed to the level of influenza A subtype and for A(H3N2) to the level of genetic subgroup. Vaccination status was based on patient (or guardian) report. Patients who self-reported receiving at least one dose of seasonal influenza vaccine ≥2 weeks before ILI onset were considered vaccinated in that season; those vaccinated <2 weeks before ILI onset were excluded. The odds of vaccination in laboratory-confirmed influenza test-positive cases was compared to influenza test-negative controls via the odds ratio (OR), derived by logistic regression and adjusted for relevant confounders. VE was derived as (1− adjusted OR) x 100%.

Age group, province, specimen collection interval and calendar time were covariates in VE analyses unless otherwise specified. Participants missing information for vaccination, timing or covariates were excluded. Age groups for covariate adjustment in primary analyses were as per usual for the SPSN (1-8, 9-19, 20-49, 50-64 and ≥65 years). Other adult age groupings (20-34, 35-54, 55-64 years) were defined for reasons specified in the main manuscript in relation to potential birth cohort (imprinting) effects. Specimen collection interval was dichotomized as ≤4 days or 5-7 days. Calendar time was based on week of specimen collection modelled using a natural cubic spline function with 3 equally spaced knots based on percentiles. In sensitivity analyses, the additional influence of sex (male/female) and comorbidity (yes/no/unknown) were explored as covariates; comorbidity includes chronic conditions that place individuals at higher risk of serious complications from influenza as defined by Canada's National Advisory Committee on Immunization (NACI) including: heart, pulmonary (including asthma), renal, metabolic (such as diabetes), blood, cancer, or immune comprising conditions; conditions that compromise management of respiratory secretions and increase risk of aspiration; or morbid obesity (body mass index ≥40). Given the late-season influenza A(H3N2) epidemic, VE estimates were also assessed with subset restriction based on epidemic period after 1 January, 10 February and 3 March 2019. To address sparse data with further stratification, VE estimates were assessed using Firth's method of penalized logistic regression (PLR), as specified [5-7].

Influenza VE – exploratory cohort analyses

To assess age-related patterns and potential cohort effects, VE was explored by single year of age among participants 1-64 years (owing to sparse data in older adults) with age smoothed as a natural cubic spline function with knots at equal percentiles of age. The number of knots (3-7) was determined by the models for each outcome with the lowest Akaike Information Criteria. Models were adjusted for province, specimen collection interval, calendar time, and age, and included an interaction term for age*vaccine status. Findings are presented for A(H3N2) overall and by genetic subgroup by age; the SPSN collects age but does not otherwise elicit birth year directly from participants. For comparison, splining models are also presented for recent prior A(H3N2) epidemics in 2016/17 and 2017/18 in Supplementary Figure S16.

Repeat vaccination effects

Repeat vaccination effects were assessed in the subset of participants ≥9 years old with complete information for current and prior seasons' vaccination status using a four-level indicator variable defined by: current season's vaccination alone; current and prior seasons' vaccination; or prior season's vaccination alone; compared to participants unvaccinated both seasons (reference group). Where data may have been sparse for current or prior seasons' vaccination alone, analysis was undertaken with subset restriction for dually vaccinated participants compared to dually unvaccinated participants.

Table S2A. Northern hemisphere influenza vaccine components, 2017/18 and 2018/19

<table>
<tr><th rowspan="2">Season</th><th colspan="2">A(H1N1)pdm09</th><th colspan="2">A(H3N2)</th><th>B(Yamagata)</th><th colspan="2">B(Victoria)</th></tr>
<tr><th>Strain (Clade)</th><th>Egg-adapted HGR</th><th>Strain (Clade)</th><th>Egg-adapted HGR</th><th>Strain[a]</th><th>Strain</th><th>Egg-adapted HGR</th></tr>
<tr><td>2017-18 (prior season)</td><td>Michigan/45/2015 (clade 6B.1)</td><td>X-275 or A/Singapore/GP1908 /2015 IVR-180</td><td>Hong Kong/4801/2014 (clade 3C.2a)</td><td>NYMC X-263B</td><td>Phuket/3073/2013 (clade 3)</td><td colspan="2">Brisbane/60/2008 (clade 1A)</td></tr>
<tr><td>2018-19 (current season)</td><td>Michigan/45/2015 (clade 6B.1)</td><td>X-275 or A/Singapore/GP1908 /2015 IVR-180</td><td>A/Singapore/INFIMH-16-0019/2016 (clade 3C.2a1)</td><td>IVR-186</td><td>Phuket/3073/2013 (clade 3)</td><td>B/Colorado/06/2017 (clade 1A(Δ2))</td><td>NYMC BX-69A</td></tr>
</table>

HGR=egg-adapted high-growth reassortant

Influenza B(Yamagata) strains shown in grey shaded cells were only included in quadrivalent influenza vaccine (QIV) formulations containing both influenza B lineages.

[a] Egg-adapted versions of B(Yamagata) are not specified or available from public sources.

Table S2B. Products by percentage of publicly-funded doses distributed, SPSN provinces, 2018/19

Product (manufacturer)[a]	Formulation (all egg-based)	Percent distributed in 2018/19
Fluviral (GSK)	TIV – Inactivated split	24%
Fluzone High-Dose (Sanofi Pasteur)	TIV – Inactivated split; 4X HA antigen	10% (Ontario only)
Influvac (BGP Pharma)	TIV – Inactivated subunit	7%
Agriflu (Novartis)	TIV – Inactivated subunit	—
Fluad (Novartis)	TIV – MF59-adjuvanted inactivated subunit	<1%
Fluzone Quadrivalent (Sanofi Pasteur)	QIV – Inactivated split	27%
Flulaval-Tetra (GSK)	QIV – Inactivated split	30%
FluMist (AstraZeneca)[b]	QIV – Live attenuated	3%

TIV=trivalent influenza vaccine; QIV=quadrivalent influenza vaccine

[a] Influenza vaccines are publicly funded for residents ≥6 months old in Alberta and Ontario and for high-risk groups and their close contacts in British Columbia (BC) and Quebec.

[b] Approved for use in individuals 2-59 years old in Canada, mostly used in children but without preferential recommendation over inactivated formulations.

Supplement S3. Laboratory methods

Nasal/nasopharyngeal specimens were collected from patients attending designated outpatient sites of the Canadian Sentinel Practitioner Surveillance Network (SPSN) using Copan flocked swabs in Universal Transport Medium^{TM} (UTM) and refrigerated until laboratory testing. All testing was performed at provincial public health reference laboratories in British Columbia (BCCDC Public Health Laboratory), Alberta (Public Health Laboratory, Alberta Precision Laboratories), Ontario (Public Health Ontario Laboratory) and Quebec (Laboratoire de santé publique du Québec). All test sites are accredited clinical and public health diagnostic and reference laboratories subject to regular proficiency testing programs, conducted at least thrice annually. Specimens were tested to the level of influenza A subtype, using in-house validated real-time reverse-transcriptase polymerase chain reaction (rRT-PCR) assays (Table S3) [8-10].

Sanger sequencing of the hemagglutinin (HA) gene was attempted on original patient specimens testing positive for influenza viruses and contributing to SPSN vaccine effectiveness analyses. Amino acid sequences were deduced from consensus nucleotide sequences with the signal peptide removed and compared to the egg-adapted vaccine high-growth reassortant (HGR) reference strains to identify amino acid substitutions at key antigenic sites according to previously published maps labelled A-E for A(H3N2) [11-13]. Antigenic sites affected by substitutions are indicated in parentheses as are substitutions affecting the receptor-binding site ("RBS") and/or associated with gain or loss of potential N-linked glycosylation ("+CHO" or "-CHO", respectively); amino acid positions previously associated with major antigenic cluster transitions were specified [13-16].

A phylogenetic tree of HA nucleotide sequences with the signal peptide removed was created using the approximate likelihood method FastTree [17] based upon a generalized time-reversible (GTR) model and visualized in FigTree [18], to determine clade and genetic sub-group distribution in conjunction with published reports [19-22]. Reference sequences were obtained from the Global Initiative on Sharing All Influenza Data (GISAID) (www.gisaid.org) [1].

Table S3. Influenza screening and subtyping assays by province

Assay	Alberta	British Columbia	Ontario	Quebec
**Influenza A/B Screen**[a] [Reference]	rRT-PCR [in-house adapted US CDC protocol]	rRT-PCR [8][b]	rRT-PCR[b] [in-house adapted US CDC protocol: [8,9]]	rRT-PCR [in-house adapted US CDC protocol]
**Influenza A subtyping**[c] [Reference]	rRT-PCR [H3: in-house adapted US CDC protocol; H1: [10]]	rRT-PCR [in-house developed; unpublished]	rRT-PCR [H3: in-house developed; H1: [9]]	rRT-PCR [in-house adapted US CDC protocol]

rRT-PCR = real time reverse-transcriptase polymerase chain reaction; US CDC = United States Centers for Disease Control and Prevention

[a] The target for influenza A virus typing was the matrix (M) gene in all provinces. The target for influenza B virus typing was the non-structural (NS1) gene in all provinces, except in British Columbia for which the influenza B target was the viral nucleoprotein (NP) gene.

[b] In BC and Ontario, the influenza virus screening rRT-PCR assay included an additional target for respiratory syncytial virus (RSV) both seasons.

[c] The target for influenza A virus subtyping in all provinces was the hemagglutinin (HA) gene. Note that the associated neuraminidase (e.g. H3N2) is inferred but not individually confirmed.

Supplement Figure S4. Epidemic curve by influenza type/subtype and week, Canadian SPSN, 2018/19

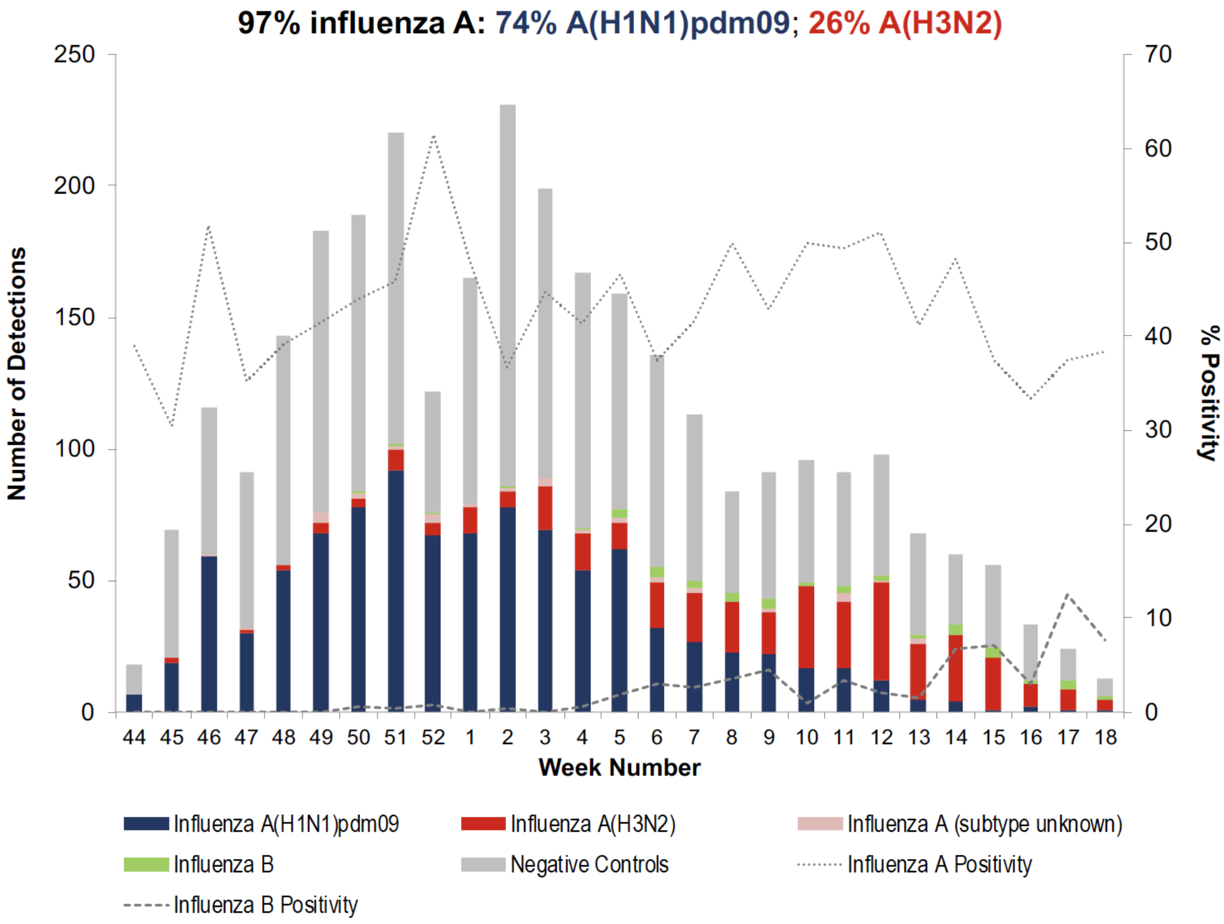

Influenza detections among 3034 specimens collected from eligible patients presenting with influenza-like illness between 1 November 2018 and 30 April 2019 (week 44 to week 18), displayed by week of specimen collection. The 2018/19 season includes 1373 influenza test positive cases and 1661 influenza test-negative controls overall. Of the test-positive cases, there were: 1332 (97%) influenza A detections and 42 (3%) influenza B detections. Of the influenza A detections there were: 969 A(H1N1)pdm09, 332 A(H3N2) and 31 A(subtype unknown), with one A(H1N1)pdm09 and A(H3N2) co-infection. Thus, among influenza A detections with known subtype, 74% (969/1301) were A(H1N1)pdm09 and 26% (332/1301) were A(H3N2). Viruses involved in the co-infection are plotted separately by type/subtype such that specimens involved in co-infection appear twice. Missing specimen collection dates were imputed as the date the specimen was received and processed at the provincial laboratory minus 2 days, the average time between the specimen collection date and laboratory received date among specimens with complete information for both values.

Supplement Table S5. Summary of influenza A(H3N2) cases by genetic subgroup

Clade with defining substitutions [antigenic site] [a]<br>+ key extra subgroup substitutions [antigenic site]	2018/19<br>N=318<br>n/N (%)
**3C.2a = 3C.2[b] + L3I + N144S [A] + F159Y [B] + K160T [B] (+CHO) + N225D (RBS) + Q311H [C] [c, d]**	-
**3C.2a1 = 3C.2a + N171K [D] [d]**	-
**3C.2a1a = 3C.2a1 + N121K [D] + T135K [A] (RBS) (-CHO) [e]**	-
**3C.2a1b = 3C.2a1 + N121K [D] + K92R [E] + H311Q [C]**	**184 (58%)**
Without T131K or T135K/N substitution [f]	-
+ T131K [A] + E62G [E] + R142G [A] [g]	145
+ T135K [A] (RBS) (-CHO) + E62G [E] + R142G [A] [f]	-
+ T135K [A] (RBS) (-CHO) + E62G [E] + T128A [B](-CHO) + R142G [A] [h]	39
+ T135N [A] (RBS) (-CHO) [f]	-
**3C.2a2 = 3C.2a + T131K [A] + R142K [A] + R261Q [E] [i]**	**21 (7%)**
**3C.2a3 = 3C.2a + N121K [D] + S144 K [A]**	**3 (<1%)**
+ T135K [A] (RBS) (-CHO) + T128A [B](-CHO) + R142G [A] + R261Q [E]	1
+ T135K [A] (RBS) (-CHO) + N126K [A] (-CHO) + R142G [A] + R150K [A] + R261Q [E]	2
**3C.2a4 = 3C.2a + N31S + D53N[C] + R142G [A] + S144R[A] + N171K [D] + I192T [B] + Q197H [B] + A304T [f, j]**	-
**3C.3a = 3C.3[k] + A138S [A] (RBS) + F159S [B] + N225D (RBS) + K326R [l]**	**110 (35%)**
+ S91N [E] + N144K [A] + F193S [B] [m]	97
+ S91N [E] + N144K [A] + F193S [B] + K82R [E] + N121D [D] [m]	13

+/- CHO = gain/loss of potential glycosylation site; RBS = substitutions affecting the receptor binding site

[a] Not all substitutions or potential sub-clusters are displayed; for additional details see phylogenetic tree in Supplement Figure S6. Dashes indicate viruses detected elsewhere but not among sequenced SPSN viruses. Note that the 2018/19 vaccine reference strain is a 3C.2a1 virus with defining substitutions in the cell-passaged consensus sequence as specified.
[b] Clade 3C.2 defined by 3C + N145S [A] + HA2: D160N.
[c] All clade 3C.2a viruses are also K160T [B] (+CHO), and P194L [B] (RBS) relative to the egg-adapted high-growth reassortant of the 2017/18 clade 3C.2a and 2018/19 clade 3C.2a1 vaccine strains and additionally S96N [D] relative to the former and G225D (RBS) relative to the latter, owing to egg-adaptation mutations in the vaccine strains (i.e. the 3C.2a vaccine is 96S, 160K and 194P and the 3C.2a1 vaccine is 160K, 194P and 225G).
[d] Clade 3C.2a and 3C.2a1 viruses without added sub-group defining substitutions were not identified among SPSN sequences in 2018/19. Their defining substitutions are displayed because detected elsewhere and because defining for other descendant viruses. Additional 3C.2a1 defining substitutions include HA2: G155E + HA2: I77V.
[e] Clade 3C.2a1a viruses were not detected among sequenced SPSN viruses in 2018/19. Defining substitutions are displayed because detected elsewhere. Additional 3C.2a1a defining substitution includes HA2: G150E.
[f] Not detected among sequenced SPSN viruses but displayed because detected elsewhere.
[g] Of these 145 clade 3C.2a1b viruses, 18 (12%) also bear Q197R [B] + S219F [D] substitutions.
[h] Of these 39 clade 3C.2a1b viruses, 3 (8%) also bear S137F [A] (RBS) + A138S [A] (RBS) + F193S [B] substitutions.
[i] Clade 3C.2a2 viruses are likely reassortants sharing the neuraminidase of 3C.2a1a, but this is not confirmed by the SPSN.
[j] Additional defining substitution includes HA2: S113A
[k] Clade 3C.3 defined by 3C + T128A[B](-CHO) + R142G [A] + N145S[A]
[l] Relative to the egg-adapted high-growth reassortant version of the 2017/18 clade 3C.2a and 2018/19 clade 3C.2a1 vaccine strains, all clade 3C.3a viruses also have P194L [B] (RBS) substitution and additionally S96N [D] relative to the former and G225D relative to the latter owing to egg-adaptation mutations in the vaccine strains (see footnote c, above). Unlike clade 3C.2a and descendant viruses, all clade 3C.3a viruses are 160K (non-glycosylated), as are the egg-adapted vaccine strains.
[m] Relative to the 2017/18 clade 3C.2a and 2018/19 clade 3C.2a1 vaccine strains, all clade 3C.3a viruses are also H311Q [C] and additionally, N144K is instead S144K and F159S is instead Y159S owing to clade-defining substitutions in 3C.2a viruses (i.e. the vaccine strains are 311H, 144S, and 159Y). Relative to the 2018/19 clade 3C.2a1 vaccine only, clade 3C.3a viruses are also K121N [D] (except where indicated to have acquired further substitution to become 121D), and K171N [D], both clade-defining substitutions of 3C.2a1 (i.e. the vaccine is 121K and 171K). Note however, that in contrast to footnote "j", clade 3C.3a viruses are not R142G relative to the 3C.2a1 vaccine because the vaccine strain acquired that parallel substitution earlier than other 3C.2a1b descendant viruses (i.e. the vaccine is also 142G, like 3C.3a viruses).

Supplement Figure S6. Phylogenetic tree, influenza A(H3N2) viruses

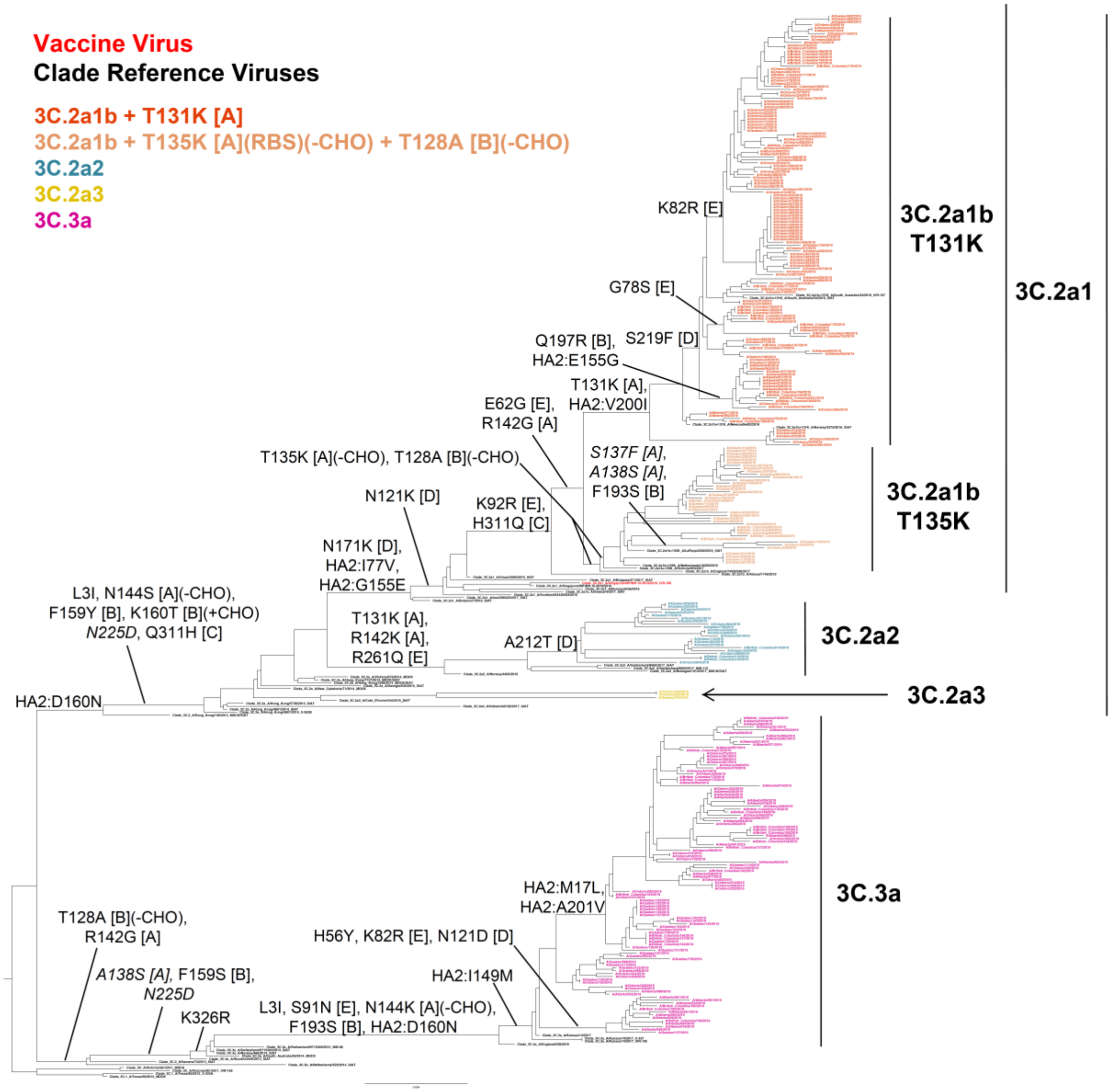

A phylogenetic tree of 314 SPSN hemagglutinin (HA) nucleotide sequences with the signal peptide removed was created using the approximate likelihood method FastTree [17] based upon a generalized time-reversible (GTR) model and visualized in FigTree [18]. Reference sequences were obtained from the Global Initiative on Sharing All Influenza Data (GISAID) (www.gisaid.org) [1]. Antigenic sites affected by specified substitutions are indicated in square brackets [], and gain or loss of potential glycosylation sites are indicated by +CHO or –CHO, respectively. Substitutions that are italicized indicate those close to or within the RBS. The HA sequences from four specimens were of insufficient quality for inclusion in the phylogenetic tree including two belonging to genetic sub-group 3C.2a1b + T131K and two belonging to clade 3C.3a.

Supplement Figure S7. Epidemic curve by influenza A(H3N2) genetic subgroup and week

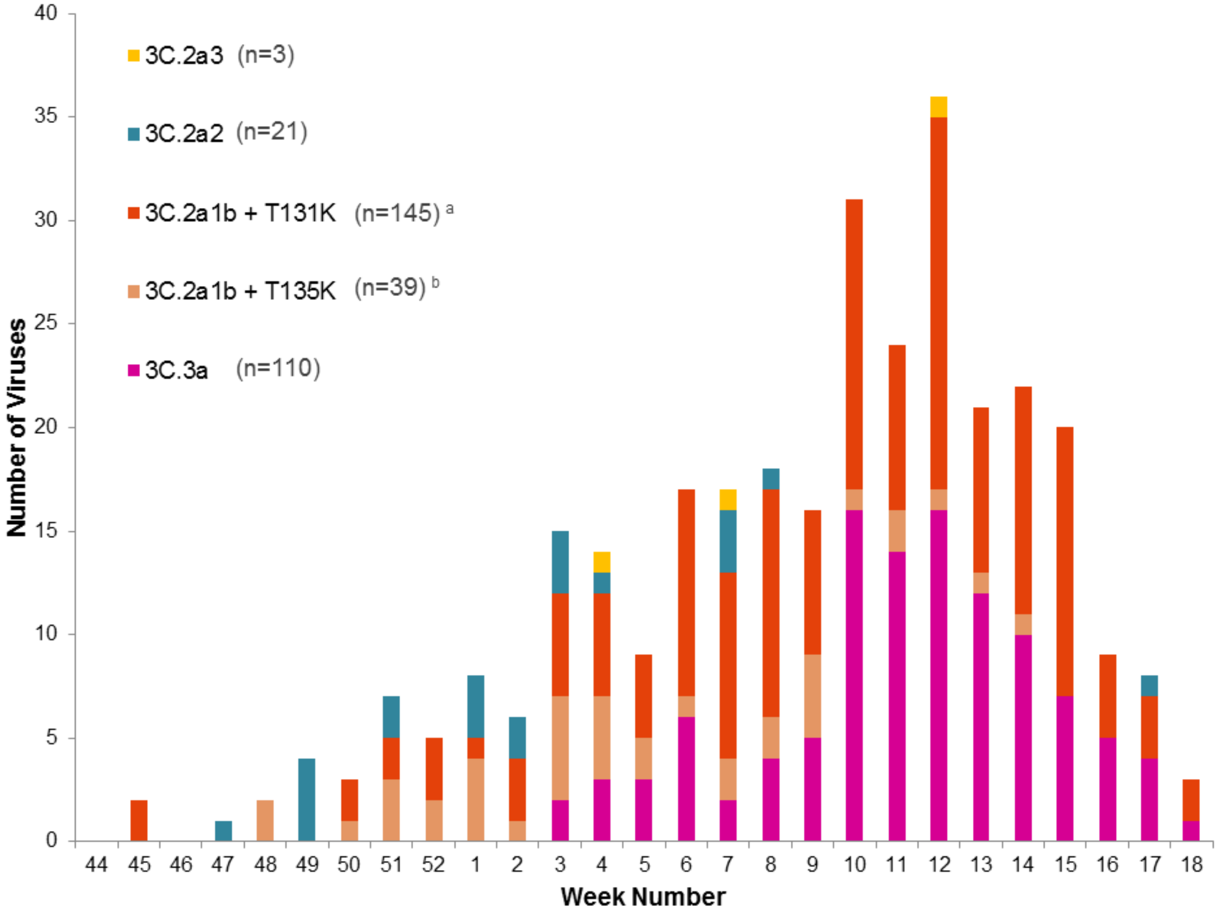

Genetic subgroup distribution of 318 influenza A(H3N2) viruses collected from patients included as cases in SPSN vaccine effectiveness analyses, displayed by week of specimen collection. Missing specimen collection dates were imputed as the date the specimen was received and processed at the provincial laboratory minus 2 days, the average time between the specimen collection date and laboratory received date among specimens with complete information for both.

[a] Of the 145 viruses belonging to subgroup 3C.2a1b with T131K [A] substitution, 18 (12%) also bore Q197R [B] + S219F [D] substitutions.

[b] Of the 39 viruses belonging to subgroup 3C.2a1b with T135K [A] substitution, all also bore E62G [E] + T128A [B](-CHO) + R142G [A] and 3/39 (8%) also bore S137F [A] (RBS) + A138S [A] (RBS) + F193S [B] substitutions.

Supplement S8. Participant characteristics Canadian SPSN, 2018/19

Distribution of participants contributing to vaccine effectiveness (VE) analyses by relevant characteristics are provided for A(H3N2) cases overall (Table S8A), and for 3C.2a1b (Table S8B) and 3C.3a cases (Table S8C), each compared to the distribution of influenza test-negative controls.

For each table, p values by case status compare the distribution of cases vs controls for the specified characteristic and p values for the proportion considered vaccinated compare the distribution of vaccinated vs unvaccinated overall by the specified characteristic. All p values are derived by chi-squared test, Fisher's exact test or Wilcoxon rank-sum test as appropriate.

Vaccination status is based on patient (or parent/guardian) report, defined as receipt of 2018/19 seasonal influenza vaccine at least 2 weeks before symptom onset. Patients vaccinated less than 2 weeks before onset of symptoms or with unknown vaccination status or timing were excluded.

Comorbidities include chronic medical conditions that place individuals at higher risk of serious complications from influenza as defined by Canada's National Advisory Committee on Immunization, including: heart, pulmonary (including asthma), renal, metabolic (such as diabetes), blood, cancer or immunocompromising conditions, conditions that compromise management of respiratory secretions and increase risk of aspiration, or morbid obesity (body mass index ≥ 40).

As in previous analyses, missing specimen collection dates were imputed as the date the specimen was received and processed at the laboratory minus 2 days, the average time between specimen collection date and laboratory received date among specimens with complete information for both values.

Table S8A. Overall influenza A(H3N2) cases and controls

	Distribution by case status (n (column %))				Proportion considered vaccinated (n (row %)) [a]			
	Overall	H3N2 Cases	Controls	p value	Overall	p value	H3N2 Cases	Controls
**N (row %)**	1993 (100)	332 (17)	1661 (83)	NA	625 (31)	0.59	100 (30)	525 (32)
**Age group (years)**								
1-8	261 (13)	41 (12)	220 (13)	0.01;<0.001[b]	60 (23)	<0.001; <0.001[b]	8 (20)	52 (24)
9-19	227 (11)	55 (17)	172 (10)		31 (14)		8 (15)	23 (13)
20-64	1247 (63)	198 (60)	1049 (63)		357 (29)		60 (30)	297 (28)
20-34	437 (22)	90 (27)	347 (21)		91 (21)		18 (20)	73 (21)
35-54	532 (27)	65 (20)	467 (28)		167 (31)		27 (42)	140 (30)
55-64	278 (14)	43 (13)	235 (14)		99 (36)		15 (35)	84 (36)
≥65	258 (13)	38 (11)	220 (13)		177 (69)		24 (63)	153 (70)
**Median (range)**	37 (1-97)	30 (1-85)	38 (1-97)	0.004	51 (1-97)	<0.001	47 (1-85)	52 (1-97)
**Sex**								
Female	1240 (62)	203 (61)	1037 (62)	0.56	433 (35)	<0.001	68 (33)	365 (35)
Male	736 (37)	128 (39)	608 (37)		186 (25)		32 (25)	154 (25)
Unknown	17 (1)	1 (0)	16 (1)	NA	6 (35)	NA	0	6 (38)
**Comorbidity**								
Overall								
No	1472 (74)	264 (80)	1208 (73)	<0.001	396 (27)	<0.001	75 (28)	321 (27)
Yes	408 (20)	45 (14)	363 (22)		204 (50)		19 (42)	185 (51)
Unknown	113 (6)	23 (7)	90 (5)	NA	25 (22)	NA	6 (26)	19 (21)
Participants aged 35-54 years								
No	403/532 (76)	48/65 (74)	355/467 (76)	0.46	118 (29)	0.01	19 (40)	99 (28)
Yes	97/532 (18)	9/65 (14)	88/467 (19)		42 (43)		5 (56)	37 (42)
Unknown	32/532 (6)	8/65 (12)	24/467 (5)	NA	7 (22)	NA	3 (38)	4 (17)
**Province**								
Alberta	444 (22)	58 (17)	386 (23)	<0.001	153 (34)	<0.001	20 (34)	133 (34)
BC	425 (21)	62 (19)	363 (22)		147 (35)		23 (37)	124 (34)
Ontario	874 (44)	149 (45)	725 (44)		286 (33)		50 (34)	236 (33)
Quebec	250 (13)	63 (19)	187 (11)		39 (16)		7 (11)	32 (17)
**Specimen collection interval from onset of influenza-like illness (ILI)**								
≤4 days	1438 (72)	285 (86)	1153 (69)	<0.001	431 (30)	0.03	85 (30)	346 (30)
5-7 days	555 (28)	47 (14)	508 (31)		194 (35)		15 (32)	179 (35)
**Median (range)**	3 (0-7)	3 (0-7)	3 (0-7)	<0.001	3 (0-7)	0.10	3 (0-7)	3 (0-7)
**Month of specimen collection**								
November	262 (13)	5 (2)	257 (15)	<0.001	50 (19)	<0.001	1 (20)	49 (19)
December	424 (21)	21 (6)	403 (24)		122 (29)		4 (19)	118 (29)
January	539 (27)	55 (17)	484 (29)		166 (31)		12 (22)	154 (32)
February	308 (15)	68 (20)	240 (14)		109 (35)		18 (26)	91 (38)
March	298 (15)	118 (36)	180 (11)		107 (36)		35 (30)	72 (40)
April	162 (8)	65 (20)	97 (6)		71 (44)		30 (46)	41 (42)

[a] The proportion self-reporting influenza vaccination, regardless of timing in relation to ILI onset was 107/339 (32%) for cases and 596/1732 (34%) for influenza test-negative controls (p=0.31).
[b] First p value is for 4 age groups (1-8, 9-19, 20-64, 65+); second p value is for 6 age groups (1-8, 9-19, 20-34, 35-54, 55-64, 65+).

Table S8B. Clade 3C.2a1b cases and controls

	Distribution by case status (n (column %))				Proportion considered vaccinated (n (row %)) [a]			
	Overall	3C.2a1b Cases	Controls	p value	Overall	p value	3C.2a1b Cases	Controls
**N (row %)**	1845 (100)	184 (10)	1661 (90)	NA	581 (32)	0.85	56 (30)	525 (32)
**Age group (years)**								
1-8	242 (13)	22 (12)	220 (13)	0.92; 0.07 [b]	55 (23)	<0.001; <0.001 [b]	3 (14)	52 (24)
9-19	192 (10)	20 (11)	172 (10)		26 (14)		3 (15)	23 (13)
20-64	1164 (63)	115 (63)	1049 (63)		330 (28)		33 (29)	297 (28)
20-34	398 (22)	51 (28)	347 (21)		83 (21)		10 (20)	73 (21)
35-54	501 (27)	34 (19)	467 (28)		152 (30)		12 (35)	140 (30)
55-64	265 (14)	30(16)	235 (14)		95 (36)		11 (37)	84 (36)
≥65	247 (13)	27 (15)	220 (13)		170 (69)		17 (63)	153 (70)
**Median (range)**	38 (1-97)	34 (1-85)	38 (1-97)	0.58	52 (1-97)	<0.001	54.5 (1-85)	52 (1-97)
**Sex**								
Female	1152 (62)	115 (62)	1037 (62)	0.89	404 (35)	<0.001	39 (34)	365 (35)
Male	677 (37)	69 (38)	608 (37)		171 (25)		17 (25)	154 (25)
Unknown	16 (1)	0	16 (1)	NA	6 (38)	NA	0	6 (38)
**Comorbidity**								
Overall								
No	1360 (74)	152 (83)	1208 (73)	0.004	364 (27)	<0.001	43 (28)	321 (27)
Yes	387 (21)	24 (13)	363 (22)		196 (51)		11 (46)	185 (51)
Unknown	98 (5)	8 (4)	90 (5)	NA	21 (21)	NA	2 (25)	19 (21)
Participants aged 35-54 years								
No	384/501 (77)	29/34 (85)	355/467 (76)	0.28	109 (28)	0.009	10 (34)	99 (28)
Yes	92/501 (18)	4/34 (12)	88/467 (19)		39 (42)		2 (50)	37 (42)
Unknown	25/501 (5)	1/34 (3)	24/467 (5)	NA	4 (16)	NA	0	4 (17)
**Province**								
Alberta	409 (22)	23 (13)	386 (23)	0.004	141 (34)	<0.001	8 (35)	133 (34)
BC	400 (22)	37 (20)	363 (22)		135 (34)		11 (30)	124 (34)
Ontario	822 (45)	97 (53)	725 (44)		269 (33)		33 (34)	236 (33)
Quebec	214 (12)	27 (15)	187 (11)		36 (17)		4 (15)	32 (17)
**Specimen collection interval from onset of influenza-like illness (ILI)**								
≤4 days	1305 (71)	152 (83)	1153 (69)	<0.001	390 (30)	0.02	44 (29)	346 (30)
5-7 days	540 (29)	32 (17)	508 (31)		191 (36)		12 (38)	179 (35)
**Median (range)**	3 (0-7)	3 (0-7)	3 (0-7)	<0.001	3 (0-7)	0.07	3 (0-7)	3 (0-7)
**Month of specimen collection**								
November	261 (14)	4 (2)	257 (15)	<0.001	50 (19)	<0.001	1 (25)	49 (19)
December	417 (23)	14 (8)	403 (24)		122 (29)		4 (29)	118 (29)
January	516 (28)	32 (17)	484 (29)		161 (31)		7 (22)	154 (32)
February	286 (16)	46 (25)	240 (14)		106 (37)		15 (33)	91 (38)
March	234 (13)	54 (29)	180 (11)		85 (37)		13 (24)	72 (40)
April	131 (7)	34 (19)	97 (6)		57 (44)		16 (47)	41 (42)

[a] The proportion self-reporting influenza vaccination, regardless of timing in relation to ILI onset was 56/184 (30%) for cases and 596/1732 (34%) for influenza test-negative controls (p=0.28).
[b] First p value is for 4 age groups (1-8, 9-19, 20-64, 65+); second p value is for 6 age groups (1-8, 9-19, 20-34, 35-54, 55-64, 65+).

Table S8C. Clade 3C.3a cases and controls

	Distribution by case status (n (column %))				Proportion considered vaccinated (n (row %)) [a]			
	Overall	3C.3a Cases	Controls	p value	Overall	p value	3C.3a Cases	Controls
**N (row %)**	1771 (100)	110 (6)	1661 (94)	NA	561 (32)	0.76	36 (33)	525 (32)
**Age group (years)**								
1-8	237 (13)	17 (16)	220 (13)	<0.001; <0.001 [b]	56 (24)	<0.001; <0.001 [b]	4 (24)	52 (24)
9-19	202 (11)	30(27)	172 (10)		28 (14)		5 (17)	23 (13)
20-64	1108 (63)	59 (54)	1049 (63)		320 (29)		23 (39)	297 (28)
20-34	374 (21)	27 (25)	347 (21)		78 (21)		5 (19)	73 (21)
35-54	488 (28)	21 (19)	467 (28)		154 (32)		14 (67)	140 (30)
55-64	246 (14)	11 (10)	235 (14)		88 (36)		4 (36)	84 (36)
≥65	224 (13)	4 (4)	220 (13)		157 (70)		4 (100)	153 (70)
**Median (range)**	37 (1-97)	23 (2-70)	38 (1-97)	<0.001	50 (1-97)	<0.001	42 (6-70)	52 (1-97)
**Sex**								
Female	1105 (62)	68 (62)	1037 (62)	0.80	390 (35)	<0.001	25 (37)	365 (35)
Male	650 (37)	42 (38)	608 (37)		165 (25)		11 (26)	154 (25)
Unknown	16 (1)	0	16 (1)	NA	6 (38)	NA	0	6 (38)
**Comorbidity**								
Overall								
No	1292 (73)	84 (76)	1208 (73)	0.07	347 (27)	<0.001	26 (31)	321 (27)
Yes	378 (21)	15 (14)	363 (22)		191 (51)		6 (40)	185 (51)
Unknown	101 (6)	11 (10)	90 (5)	NA	23 (23)	NA	4 (36)	19 (21)
Participants aged 35-54 years								
No	367/488 (75)	12/21 (57)	355/467 (76)	0.99	108 (29)	0.01	9 (75)	99 (28)
Yes	91/488 (19)	3/21 (14)	88/467 (19)		39 (43)		2 (67)	37 (42)
Unknown	30/488 (6)	6/21 (29)	24/467 (5)	NA	7 (23)	NA	3 (50)	4 (17)
**Province**								
Alberta	420 (24)	34 (31)	386 (23)	<0.001	145 (35)	<0.001	12 (35)	133 (34)
BC	383 (22)	20 (18)	363 (22)		136 (36)		12 (60)	124 (34)
Ontario	756 (43)	31 (28)	725 (44)		245 (32)		9 (29)	236 (33)
Quebec	212 (12)	25 (23)	187 (11)		35 (17)		3 (12)	32 (17)
**Specimen collection interval from onset of influenza-like illness**								
≤4 days	1255 (71)	102 (93)	1153 (69)	<0.001	380 (30)	0.05	34 (33)	346 (30)
5-7 days	516 (29)	8 (7)	508 (31)		181 (35)		2 (25)	179 (35)
**Median (range)**	3 (0-7)	3 (0-7)	3 (0-7)	<0.001	3 (0-7)	0.1	2 (0-5)	3 (0-7)
**Month of specimen collection**								
November	257 (15)	0	257 (15)	<0.001	49 (19)	<0.001	0	49 (19)
December	403 (23)	0	403 (24)		118 (29)		0	118 (29)
January	492 (28)	8 (7)	484 (29)		155 (32)		1 (13)	154 (32)
February	255 (14)	15 (14)	240 (14)		94 (37)		3 (20)	91 (38)
March	241 (14)	61 (56)	180 (11)		94 (39)		22 (36)	72 (40)
April	123 (7)	26 (24)	97 (6)		51 (41)		10 (39)	41 (42)

[a] The proportion self-reporting influenza vaccination, regardless of timing in relation to ILI onset was 36/110 (33%) for cases and 596/1732 (34%) for influenza test-negative controls (p=0.72).
[b] First p value is for 4 age groups (1-8, 9-19, 20-64, 65+); second p value is for 6 age groups (1-8, 9-19, 20-34, 35-54, 55-64, 65+).

Supplement Table S9. Influenza A(H3N2) vaccine effectiveness overall and by genetic subgroup, Canadian SPSN, 2018/19

Model and covariates	Controls n vac/ N total	A(H3N2)			3C.2a1b			3C.3a		
		Cases n vac/N	Unadjusted VE % (95% CI)	Adjusted VE % (95% CI)	Cases n vac/N	Unadjusted VE % (95% CI)	Adjusted VE % (95% CI)	Cases n vac/N	Unadjusted VE % (95% CI)	Adjusted VE % (95% CI)
Age group[a], province[b], interval[c], calendar time[d]	525/1661	100/332	7 (-21 to 28)	17 (-13 to 39)	56/184	5 (-32 to 32)	27 (-7 to 50)	36/110	-5 (-59 to 30)	-32 (-119 to 21)
+ Comorbidity [a, e, f]				14 (-18 to 37)			24 (-11 to 49)			-39 (-131 to 17)
Restricted week 1 to week 18 [a, e, g]	367/1024	95/307	20 (-5 to 39)	17 (-15 to 39)	51/167	21 (-12 to 45)	33 (-1 to 55)	36/110	13 (-32 to 43)	-31 (-117 to 21)
Restricted week 7 to week 18 [a, e, g]	175/423	78/233	29 (0 to 49)	17 (-21 to 43)	40/122	31 (-6 to 55)	37 (-1 to 61)	34/96	22 (-23 to 51)	-54 (-172 to 13)
Restricted week 10 to week 18 [a, e, g]	111/273	65/180	18 (-22 to 44)	-4 (-62 to 34)	29/87	27 (-21 to 56)	34 (-18 to 63)	32/85	12 (-45 to 47)	-90 (-256 to -2)
**Restricted by age subsets**										
1-19 years [e,h]	75/392	16/96	15 (-53 to 53)	48 (-5 to 74)	6/42	30 (-73 to 71)	52 (-25 to 82)	9/47	0 (-116 to 54)	43 (-44 to 77)
+ Comorbidity [e, f, h]				49 (-3 to 75)			53 (-14 to 83)[i]			47 (-36 to 79)
20-64 years [e, j]	297/1049	60/198	-10 (-53 to 21)	-7 (-56 to 26)	33/115	-2 (-56 to 33)	6 (-49 to 41)	23/59	-62 (-178 to 6)	-96 (-277 to -2)
+ Comorbidity [e, f, j]				-13 (-65 to 23)			0 (-60 to 37)			-100 (-289 to -3)
20-34 years [e]	73/347	18/90	6 (-67 to 47)	0 (-89 to 48)	10/51	8 (-91 to 56)	5 (-112 to 57)	5/27	15 (-133 to 69)	-21 (-283 to 62)
+ Comorbidity [e, f]				-1 (-93 to 47)			2 (-120 to 56)			-16 (-269 to 64)
35-54 years [e]	140/467	27/65	-66 (-182 to 2)	-68 (-207 to 8)	12/34	-27 (-165 to 39)	-20 (-163 to 46)	14/21	-367 (-1082 to -85)	-346 (-1321 to -58) [i]
+ Comorbidity [e, f]				-80 (-231 to 3)			-25 (-177 to 43)			-433 (-1820 to -72) [i]
55-64 years [e]	84/235	15/43	4 (-90 to 51)	35 (-44 to 71)	11/30	-4 (-129 to 53)	26 (-80 to 70)	4/11	-3 (-261 to 71)	30 (-180 to 84) [i]
+ Comorbidity [e, f]				32 (-54 to 70)			20 (-101 to 68)			30 (-176 to 84) [i]
65 + years [e]	153/220	24/38	25 (-54 to 63)	62 (9 to 84)	17/27	26 (-71 to 68)	61 (-4 to 85)	4/4	NE	NE
+ Comorbidity [e, f]				63 (9 to 85)			63 (2 to 86) [i]			NE
**Adjusted for sex**										
Overall [a, e, k]	519/1645	100/331	6 (-21 to 27)	16 (-15 to 38)	56/184	5 (-32 to 32)	26 (-9 to 50)	36/110	-6 (-59 to 30)	-32 (-119 to 21)
+ Comorbidity [a, e, f, k]				12 (-20 to 36)			23 (-13 to 48)			-39 (-132 to 17)

VE = vaccine effectiveness; Vac = vaccinated; 95%CI = 95% confidence interval; NE = Not estimable – model does not converge

[a] Adjusted for age group specified as 1-8, 9-19, 20-49, 50-64 and ≥ 65 years
[b] Adjusted for province specified as: Alberta, British Columbia, Ontario, Quebec.
[c] Adjusted for specimen collection interval specified as: ≤4 days; 5-7 days.
[d] Adjusted for calendar time based on week of specimen collection modeled using natural cubic spline function with 3 equally spaced knots based on percentiles.
[e] Adjusted for province, specimen collection interval and calendar time unless otherwise specified.
[f] Adjusted for comorbidity as yes/no/unknown. Comorbidity includes chronic conditions that place individuals at higher risk of serious complications from influenza as defined by Canada's National Advisory Committee on Immunization (NACI) including: heart, pulmonary (including asthma), renal, metabolic (such as diabetes), blood, cancer, or immune comprising conditions; conditions that compromise management of respiratory secretions and increase risk of aspiration; or morbid obesity (body mass index ≥40).
[g] Detection rates were 10% or higher for specimen collection dates between weeks 7-18 (10 February – 30 April 2019) for 3C.2a1b, and weeks 10-17 (3 March – 27 April 2019) for 3C.3a; overall, 48% (88/184) 3C.2a1b but 79% (87/110) 3C.3a viruses were detected from 1 March 2019.
[h] Adjusted for age group specified as 1-8 and 9-19 years
[i] Firth's method of penalized logistic regression used due to limited sample size.
[j] Adjusted for age group specified as 20-49 years and 50-64 years
[k] Additionally adjusted for sex as female/male (excluding unknown sex).

Table S9 Cont'd. VE against predominant 3C.2a1b subgroup with T131K substitution

Model and covariates	Controls n vacc / N total	3C.2a1b with T131K substitution		
		Cases n vacc / N total	Unadjusted VE % (95% CI)	Adjusted VE % (95% CI)
Age group [a], province [b], interval [c], calendar time [d]	525/1661	43/145	9 (-32 to 37)	37 (3 to 59)
+ Comorbidity [a, e, f]				35 (0 to 58)
**Restricted by age sub-sets**				
1-19 years [e, g]	75/392	5/34	27 (-95 to 73)	51 (-38 to 83)
20-64 years [e, h]	297/1049	24/91	9 (-47 to 44)	29 (-21 to 58)
20-34 years [e]	73/347	7/43	27 (-71 to 69)	30 (-76 to 72)
35-54 years [e]	140/467	8/26	-4 (-144 to 56)	24 (-91 to 70)
55-64 years [e]	84/235	9/22	-24 (-203 to 49)	32 (-86 to 75)
65 + years [e]	153/220	14/20	-2 (-177 to 62)	50 (-63 to 84)

VE = vaccine effectiveness; Vac = vaccinated; 95%CI = 95% confidence interval; NE = Not estimable – model does not converge

[a] Adjusted for age group specified as 1-8, 9-19, 20-49, 50-64 and ≥ 65 years
[b] Adjusted for province specified as: Alberta, British Columbia, Ontario, Quebec
[c] Adjusted for specimen collection interval specified as: ≤4 days; 5-7 days
[d] Adjusted for calendar time based on week of specimen collection modeled using natural cubic spline function with 3 equally spaced knots
[e] Adjusted for province, specimen collection interval and calendar time
[f] Adjusted for comorbidity as yes/no/unknown. Comorbidity includes chronic conditions that place individuals at higher risk of serious complications from influenza as defined by Canada's National Advisory Committee on Immunization (NACI) including: heart, pulmonary (including asthma), renal, metabolic (such as diabetes), blood, cancer, or immune comprising conditions; conditions that compromise management of respiratory secretions and increase risk of aspiration; or morbid obesity (body mass index ≥40)
[g] Adjusted for age group specified as 1-8 and 9-19 years
[h] Adjusted for age group specified as 20-49 years and 50-64 years

Supplement Table S10. Repeat vaccination effects: influenza A(H3N2) overall and by genetic clade

Table S10A. Stratified analysis - 4-level indicator variable

**Model and outcomes** [a, b] (4-level indicator variable)	**N Controls**	**A(H3N2)**			**3C.2a1b**			**3C.3a**		
		**N Cases**	**Unadjusted VE % (95% CI)**	**Adjusted VE % (95% CI)**	**N Cases**	**Unadjusted VE % (95% CI)**	**Adjusted VE % (95% CI)**	**N Cases**	**Unadjusted VE % (95% CI)**	**Adjusted VE % (95% CI)**
Unvaccinated both 2017/18 and 2018/19 seasons	704	158	Ref	Ref	87	Ref	Ref	48	Ref	Ref
Vaccinated prior (2017/18) season only	169	21	45 (10 to 66)	33 (-13 to 61)	11	47 (-1 to 72)	34 (-30 to 66)	7	36 (-33 to 73)[c]	21 (-88 to 70)[c]
Vaccinated current (2018/19) season only	55	5	59 (-3 to 84)	69 (18 to 89)	4	41 (-66 to 79)	59 (-21 to 86)	0	87 (6 to 100)[c]	89 (9 to 100)[c]
Vaccinated both 2017/18 and 2018/19 seasons	376	83	2 (-32 to 27)	-1 (-45 to 29)	47	-1 (-47 to 31)	14 (-34 to 45)	30	-18 (-87 to 27)[c]	-121 (-313 to -19)[c]

Table S10B. Stratified analysis - 2-level subset restriction

**Model and outcomes** [a, b, d] (subset restriction)	**N Controls**	**A(H3N2)**			**3C.2a1b**			**3C.3a**		
		**N Cases**	**Unadjusted VE % (95% CI)**	**Adjusted VE % (95% CI)**	**N Cases**	**Unadjusted VE % (95% CI)**	**Adjusted VE % (95% CI)**	**N Cases**	**Unadjusted VE % (95% CI)**	**Adjusted VE % (95% CI)**
Unvaccinated both 2017/18 and 2018/19 seasons	704	158	Ref	Ref	87	Ref	Ref	48	Ref	Ref
Vaccinated both 2017/18 and 2018/19 seasons	376	83	2 (-32 to 27)	1 (-42 to 31)	47	-1 (-47 to 31)	15 (-33 to 46)	30	-17 (-88 to 27)	-115 (-304 to -15)

VE = vaccine effectiveness; 95%CI = 95% confidence interval

[a] Same exclusion criteria as primary analysis; additionally restricted to participants ≥9 years old and with complete data for current and prior season's vaccine receipt.
[b] Adjusted for age group (9-19, 20-49, 50-64, ≥ 65 years), province (Alberta, British Columbia, Ontario, Quebec), specimen collection interval (≤4 days; 5-7 days) and calendar time (week of specimen collection modeled using natural cubic spline function with 3 equally spaced knots) unless otherwise specified.
[c] Firth's method of penalized logistic regression used due to limited sample size
[d] Subset restriction to participants known to be vaccinated or unvaccinated both seasons.

Supplement Figure S11. Percentage histogram of vaccinated influenza A(H3N2) cases and controls by single year of age, 2014/15, 2016/17, and 2017/18 epidemics

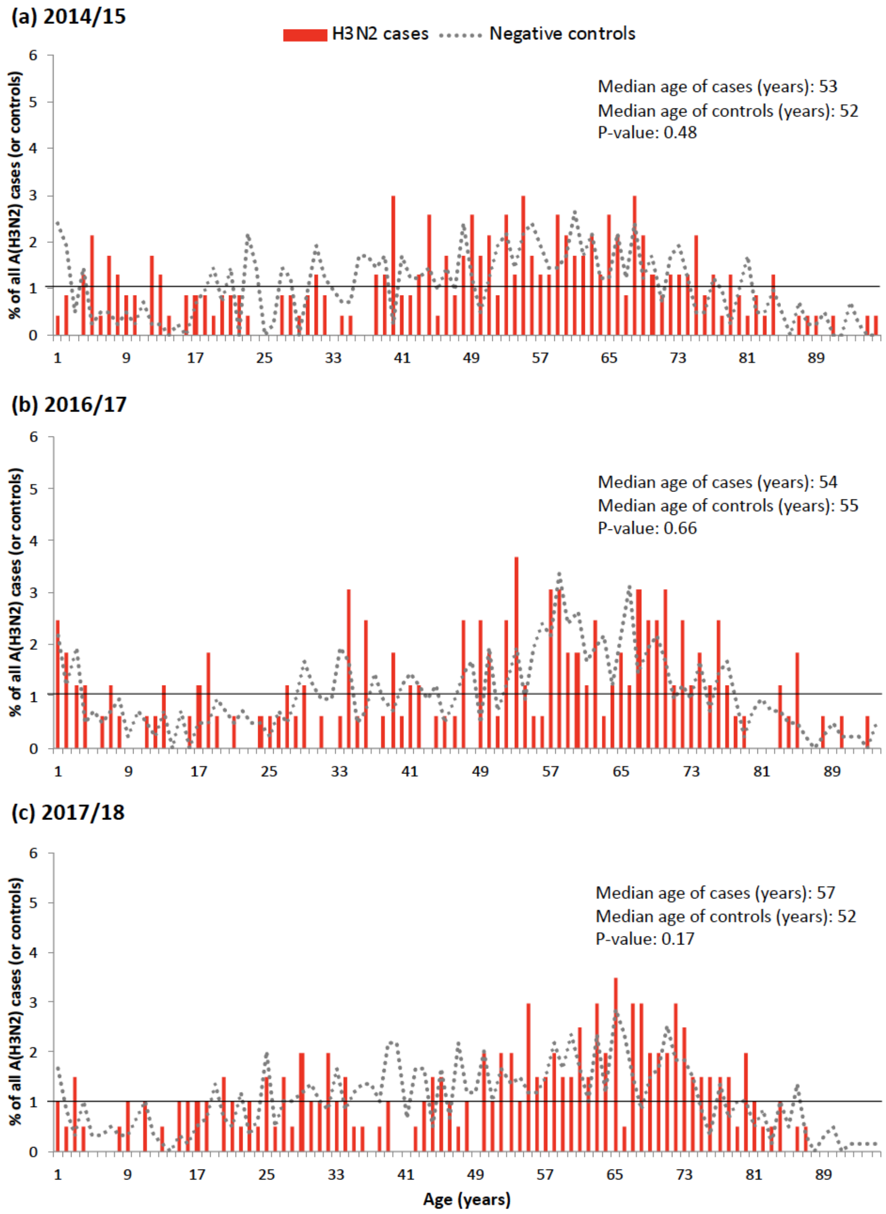

Age distributions of vaccinated participants are displayed for influenza A(H3N2) cases and influenza test-negative controls identified by the SPSN between November and April during seasonal epidemics in 2014/15 (panel a: 234 vaccinated cases; 415 vaccinated controls); 2016/17 (panel b: 163 vaccinated cases; 416 vaccinated controls) and 2017/18 (panel c: 201 vaccinated cases; 596 vaccinated controls). The percentage of all influenza A(H3N2) cases belonging to a given single year of age is displayed as a red bar; the same information for test-negative controls is superimposed as a dotted line to indicate the sampling distribution by age for comparison purposes. The p values displayed are for the comparison between median ages of cases and controls within the same season. Vaccination status is based on self-report (or by parent/guardian) of at least one dose of influenza vaccine ≥2 weeks prior to onset of influenza-like illness (ILI) as applied for vaccine effectiveness (VE) analyses. Participants vaccinated <2 weeks before ILI onset are excluded.

Supplement Table S12. Amino-acid variation at select antigenic sites of historic influenza A(H3N2) viruses since 1968, aligned by 5-year age bands and corresponding birth cohorts reflecting potential priming epochs

Select HA residue [antigenic site]	2018/19 vaccine and A(H3N2) genetic subgroups			Five-year age groupings and corresponding birth cohorts (derived as 2018 - age in years) with associated amino acids and glycosylation (CHO) sites at select residues of historic A(H3N2) viruses that were prevalent and/or may have served as A(H3N2) priming infections during the specified period (i.e. potential priming epochs)											
	3C.2a1 vaccine (egg-adapted) *a*	3C.2a1b	3C.3a	55+ 1963 -	50-54 1964-68	45-49 1969-73	40-44 1974-78	35-39 1979-83	30-34 1984-88	25-29 1989-93	20-24 1994-98	15-19 1999-03	10-14 2004-08	5-9 2009-13	1-4 2014-17
45 [C]	**N (CHO)**	**N (CHO)**	**N (CHO)**	—	S	S	S	S	S	S	S	S	S	**S/N (+/- CHO)**	**N (CHO)**
62 [E]	E	G	E	—	I	I	I/K	K	K	K	K/E	E	E	E	E
63 [E]	**N (CHO)**	**N (CHO)**	**N (CHO)**	—	**N (+ CHO)**	**D/N (+ CHO)**	**D/N (+ CHO)**	**N (CHO)**	**N (CHO)**	**N (CHO)**	**N (CHO)**	**N (CHO)**	**N (CHO)**	**N (CHO)**	**N (CHO)**
81 [E]	**N**	**N**	**N**	—	**N (+ CHO)**	**D/N (CHO)**	N	N	N	N	N	N	N	N	N
91 [E]	S	S	**N**	—	S	S	S	S	S	S	S	S	S	S	S
92 [E]	K	R	K	—	K	K	K	K	K	K	K/T	K/T	K	K	K/R
96 [D]	N *b*	N	N	—	N	N	N	N	N	N	N	N	N	N	N
121 [D]	K	K	**N**	—	I	I	I	I	I	I/T	I/T/N	**N**	**N**	**N**	N/K
122 [A]	N *(CHO)*	N *(CHO)*	N *(CHO)*	—	T	T/S/N	N	N	N	N	N *(+ CHO)*	N *(CHO)*	N *(CHO)*	N *(CHO)*	N *(CHO)*
126 [A]	**N (CHO)**	**N (CHO)**	**N**	—	T	T	**N (+ CHO)**	**N (CHO)**	**N (CHO)**	**N (CHO)**	**N (CHO)**	**N (CHO)**	**N (CHO)**	**N (+/-CHO)**	**N (+/-CHO)**
128 [B]	T	T/A *c*	**A (-CHO)**	—	T	T	T	T	T	T	T	T	T	T/A	T/A
131 [A]	T	K/T *d*	T	—	T	T	T	T	T	A	A	A/T	T	T	T/K
**133 [A]** *e*	**N (CHO)**	**N (+/- CHO)**	**N (CHO)**	—	N	N	N/S	S	S	S/D	**D/N (+/- CHO)**	**N (+CHO)**	**N (CHO)**	**N (CHO)**	**N (CHO)**
**134 (RBS)**	G	G	G	—	G	G	G	G	G	G	G	G	G	G	G
**135 [A] (RBS)** *e*	T	T/K *d*	T	—	G	G	G	G	G	G/E/K	K/T	T	T	T	T
**138 [A] (RBS)**	A	A	**S**	—	A	A	A	A	A	A	A	A	A	A	A
142 [A]	G	G	G	—	G	G	G	G	G	G	G/R	R	R	R/G	R/G/K
144 [A]	S *(- CHO)*	S *(- CHO)*	**K *(- CHO)***	—	G	G/D	D	D/G	V	V	V/I	**I/D/N (+ CHO)**	**N (CHO)**	**N (CHO)**	N/S *(+/- CHO)*
**145 [A]** *f*	S	S	S	—	S	S	S/N	N	N	N/K	K/N	K	N	N/S	S
**155 [A] (RBS)** *f*	T	T	T	—	T	T/Y	Y	Y	Y/H	H	H	H/T	T	T	T
**156 [B]** *f*	H	H	H	—	K	K	K	E	E	E/K	K/Q	Q	H	H	H
**158 [B]** *f*	N	**N (CHO)**	N	—	G	G	G/E	E	E	E/D	E/K	K	K	N	**N (+ CHO)**
**159 [B]** *f*	**Y**	**Y**	**S**	—	**S**	**S**	**S**	**S**	S/Y	Y	Y	Y	F	F	Y/S
160 [B]	**K (- CHO)** *g, h*	**T (CHO)** *h*	K *i*	—	T *j*	T *j*	T *j*	K *k*	K *k*	K *k*	K *k*	K *k*	K *k*	K *k*	T
165 [B]	**N (CHO)**	**N (CHO)**	**N (CHO)**	—	**N (+ CHO)**	**N (+ CHO)**	**N (CHO)**	**N (CHO)**	**N (CHO)**	**N (CHO)**	**N (CHO)**	**N (CHO)**	**N (CHO)**	**N (CHO)**	**N (CHO)**
171 [D]	K	K	**N**	—	**N**	**N**	**N**	**N**	**N**	**N**	**N**	**N**	**N**	**N**	N/K
**189 [B]** *f*	K	K	K	—	Q	Q	Q/K	K	K/R	R/S	S	S	N	K	K
**193 [B]** *e, f*	F	F	**S**	—	**S**	**S**	N/D	N	N	N/S	**S**	S	S/F	F	F
**194 [B] (RBS)**	P *g*	L	L	—	L	L	L	L	L	L	I	L	L	L	L
**225 (RBS)**	G *l*	D	D	—	G	G	G	G	G	G	G	G/D	D/N	N	D
246 [D]	**N (CHO)**	**N (CHO)**	**N (CHO)**	—	N	N	N	**N (+ CHO)**	**N (CHO)**	**N (CHO)**	**N (CHO)**	**N (CHO)**	**N (CHO)**	**N (CHO)**	**N (CHO)**
311 [C]	H	Q	Q	—	Q	Q	Q	Q	Q	Q	Q	Q	Q	Q	H/Q
**Number of A(H3N2) glycosylation sites (CHO):**				—	3	1-3	2-3	4	4	4	4-6	5-7	6-7	5-8	6-9

HA: hemagglutinin; **CHO:** confirmed glycosylation site; italicized *(CHO)* means determined in silico (<0.5) (does not pass threshold); (+/- CHO) means gain/loss of glycosylation

Amino acid variation at 32 hemagglutinin (HA) antigenic site positions were compared between 83,026 historic A(H3N2) viruses since 1968, including the 2018/19 egg-adapted 3C.2a1 vaccine and contemporary 3C.3a and 3C.2a1b circulating viruses, based on A(H3) sequences downloaded on 31 July 2019 from the Global Initiative on Sharing All Influenza Data (GISAID) [1]. This is shown grouped by 5-year age bands and corresponding birth cohorts, with the range of birth years derived as (2018 – age, in years) to reflect potential priming epochs.

A thicker border is drawn around the cohort 35-54 years old in 2018/19 in whom a signal of pre-existing protection against clade 3C.3a viruses was suggested among unvaccinated participants but a signal of increased risk among vaccinated participants. Note that A(H3N2) viruses became prevalent following the 1968 pandemic. Adults 55+ years of age in 2018/19 were likely primed with A(H2N2) viruses. Adults 50-54 years of age in 2018/19 may have been primed with A(H2N2) or A(H3N2) viruses but we have assumed a 4-5 year delay from birth to first influenza infection and A(H3N2) priming on that basis. Children <5 years of age in 2018/19 may not have yet had a first influenza priming exposure.

Colour shading legend and footnotes overleaf.

Colour Shading Legend

**Residue [antigenic site]** *f*	Residues [in specified antigenic site] that are also major antigenic cluster transition positions as per Koel et al [13]. See also footnote *f*.
**Residue [antigenic site] (RBS)**	Residues [in specified antigenic site] located in or adjacent to the receptor binding site (RBS). May also be a major antigenic cluster transition position, or accessory position, as per Koel et al [13] if associated with footnotes *e* and f.
Amino acid	Amino acids at the specified residue found in the 2018/19 vaccine and, where indicated by colour shading, shared with 2018/19 circulating 3C.2a1b and 3C.3a viruses
Amino acid	Amino acids at the specified residue found in 2018/19 circulating 3C.2a1b and 3C.3a viruses but not in the 2018-19 vaccine
Amino acid	Amino acid (or glycosylation) in 2018/19 circulating 3C.2a1b viruses but not in the 2018/19 vaccine or in 3C.3a viruses
**Amino acid**	Amino acid in 2018/19 circulating 3C.3a viruses (or historic viruses) but not in the 2018/19 vaccine or in 3C.2a1b viruses

Footnotes:

a. The 2018-19 clade 3C.2a1 vaccine is distinguished from the preceding 2016/17 and 2017/18 clade 3C.2a vaccine strains by N121K [D], N171K [D] and R142G [A] antigenic site substitutions. Otherwise, residues in the 2016/17, 2017/18 and 2018/19 vaccine strains are shared. The egg-adapted versions additionally share two of three mutations in common, including T160K [B] (-CHO) and L194P [B](RBS). The third egg-adaptation substitution in the 2016/17 and 2017/18 vaccine is N96S [D] and in the 2018/19 3C.2a1 vaccine is D225G (RBS).

b. Egg adaptation mutation in the 2016/17 and 2017/18 clade 3C.2a vaccines (S96) but not the 2018/19 clade 3C.2a1 vaccine (N96).

c. During the 2018/19 season, 79% of 3C.2a1b viruses sequenced by the SPSN were T128 and 21% were A128 (the latter in conjunction with T135K-bearing viruses).

d. During the 2018/19 season, 79% of 3C.2a1b viruses sequenced by the SPSN were K131 and 21% were T131; conversely 79% of 3C.2a1b viruses were T135 and 21% were K135.

e. An accessory antigenic cluster transition position as per Koel et al [13].

f. A major antigenic cluster transition position as per Koel et al [13].

g. Egg adaptation mutation in the 2016/17 and 2017/18 clade 3C.2a vaccines and the 2018/19 clade 3C.2a1 vaccine.

h. Circulating 3C.2a and descendant viruses (e.g. 3C.2a1 and 3C.2a1b) in 2016/17, 2017/18 and 2018/19 were 160T (+ CHO). The egg-adapted 3C.2a and 3C.2a1 vaccines have lost the glycosylation site (-CHO) at position 160 with egg adaptation. Adjacent residues are shielded from antibody and other immune system access in wild-type viruses but exposed in egg-adapted strains.

i. Like the egg adapted 3C.2a and 3C.2a1 vaccine strains, 3C.3a viruses are not glycosylated because a lysine (K) rather than a threonine (T) at position 160; the 159 and other adjacent residues are therefore not shielded by glycosylation (i.e. are exposed to antibody and other immune system interaction) in 3C.3a viruses.

j. These historic viruses are a threonine (T) at position 160 (like 3C.2a1b viruses), but unlike 3C.2a1b viruses were not glycosylated because position 158 is not asparagine (N). The 159 and other adjacent residues are exposed (i.e. are not shielded by glycosylation from antibody or other immune system interaction).

k. A lysine (K) and not glycosylated: the 159 and adjacent residues are not shielded by glycosylation from antibody or other immune system interaction.

l. Egg adaptation mutation in the 2018/19 3C.2a1 vaccine (G225) but not the 2016/17 or 2017/18 3C.2a vaccines (D225).

Supplement Table S13. Global influenza A(H3) position 159 and 193 amino acid tallies, by year, 1968 to 2019

Position 159	1968	1969	1970	1971	1972	1973	1974	1975	1976	1977	1978	1979	1980	1981	1982	1983	1984	1985	1986	1987	1988	1989	1990	1991	1992	1993	1994	1995	1996	1997	1998	1999	2000	2001	2002	2003	2004	2005	2006	2007	2008	2009	2010	2011	2012	2013	2014	2015	2016	2017	2018	2019	Total
Total	112	12	10	15	33	14	18	21	26	21	9	13	23	18	31	32	32	48	17	35	31	105	63	84	142	231	149	196	271	211	308	370	422	323	876	1585	1348	1490	1297	2026	2085	2269	1928	2481	4002	3099	5990	7775	8880	15524	9840	7085	83026
Alanine (A)																																																					
Cysteine (C)					1															1																																	2
Aspartic Acid (D)																				1						1	1	1			1			1	1										1								8
Phenylalanine (F)																												2							6	127	1126	1464	1277	2022	2020	2172	1912	2449	3821	3019	2376	934	50	84	7	9	24877
Glycine (G)																																									1												1
Histidine (H)																				1		7	1		1				1							2												1	1	1	1		17
Isoleucine (I)																																																					
Lysine (K)																																																					
Leucine (L)																																																					
Asparagine (N)				1			2	4	1											1		2	3			3	1					5	1	2	7	54						14		1	1				3				106
Glutamine (Q)																				1																																	1
Arginine (R)				3	2																																																5
Serine (S)	110	12	9	11	28	14	15	17	25	21	9	13	23	18	31	32	32	26	6	1				1	1	1	1										2		1	1	56	62			8	26	953	577	1062	467	863	3091	7626
Threonine (T)							1																																														1
Tyrosine (Y)			1															21	11	28	31	96	57	83	140	226	146	193	270	211	306	365	420	320	862	1400	216	22	1	2		13	15	21	165	46	2610	6219	7754	14956	8961	3961	50149
Unknown (X)	2				2													1		1			2								1		1			2	4	4	18	1	8	8	1	10	6	8	51	44	10	16	8	24	233

Position 193	1968	1969	1970	1971	1972	1973	1974	1975	1976	1977	1978	1979	1980	1981	1982	1983	1984	1985	1986	1987	1988	1989	1990	1991	1992	1993	1994	1995	1996	1997	1998	1999	2000	2001	2002	2003	2004	2005	2006	2007	2008	2009	2010	2011	2012	2013	2014	2015	2016	2017	2018	2019	Total
Total	112	12	10	15	33	14	18	21	26	21	9	13	23	18	31	32	32	48	17	35	31	105	63	84	142	231	149	196	271	211	308	370	422	323	876	1585	1348	1490	1297	2026	2085	2269	1928	2481	4002	3099	5990	7775	8880	15524	9840	7085	83026
Alanine (A)																																									1												1
Cysteine (C)																																																1					1
Aspartic Acid (D)								7	21	9					1																																						38
Phenylalanine (F)																																			2	2	19	660	1111	2009	2027	2238	1889	2425	3833	3040	5910	7687	8591	14640	8798	3753	68634
Glycine (G)																																				3	2	1													1		7
Histidine (H)																																																					
Isoleucine (I)																											2									1						2			1	1	2			1			10
Lysine (K)								1		1		2	1		3	1		3	1	5	1	7	4		1																		1										32
Leucine (L)																																											1				6	4		8	4	2	25
Asparagine (N)	2		1	2	3	4	16	9	4	10	9	11	21	18	27	31	32	44	16	29	30	92	25	6	1	2	1		2	1	1	3		3		100	31	13	9	1		3	9	20	148	33	4	2	2	1			832
Glutamine (Q)																																																					
Arginine (R)																							2	1	6	2	5	11	18	4	2		1		1	3	6	5															67
Serine (S)	108	12	9	13	28	10	2	4	1	1												6	30	77	134	227	141	185	250	205	305	367	420	320	873	1475	1286	810	172	12	56	21	3	20	8	15	43	45	276	846	1023	3327	13166
Threonine (T)																													1																								1
Tyrosine (Y)																																							2				8	2	2	3	5	2	3	2	1		30
Unknown (X)	2				2								1					1		1			2							1			1			1	4	1	3	4	1	5	17	14	10	7	20	34	8	26	13	3	182

Tally of amino acids at hemagglutinin (HA) position 159 and 193 among 83,026 influenza A(H3N2) sequences with specimen collection dates from 1 January 1968 to 31 July 2019 downloaded from the Global Initiative on Sharing All Influenza Data (GISAID) [1] and aligned using MAFFT v7.245 [23].

Supplement Figure S14. Percentage histogram of clade 3C.2a1b and clade 3C.3a cases and controls by age subset and vaccination status

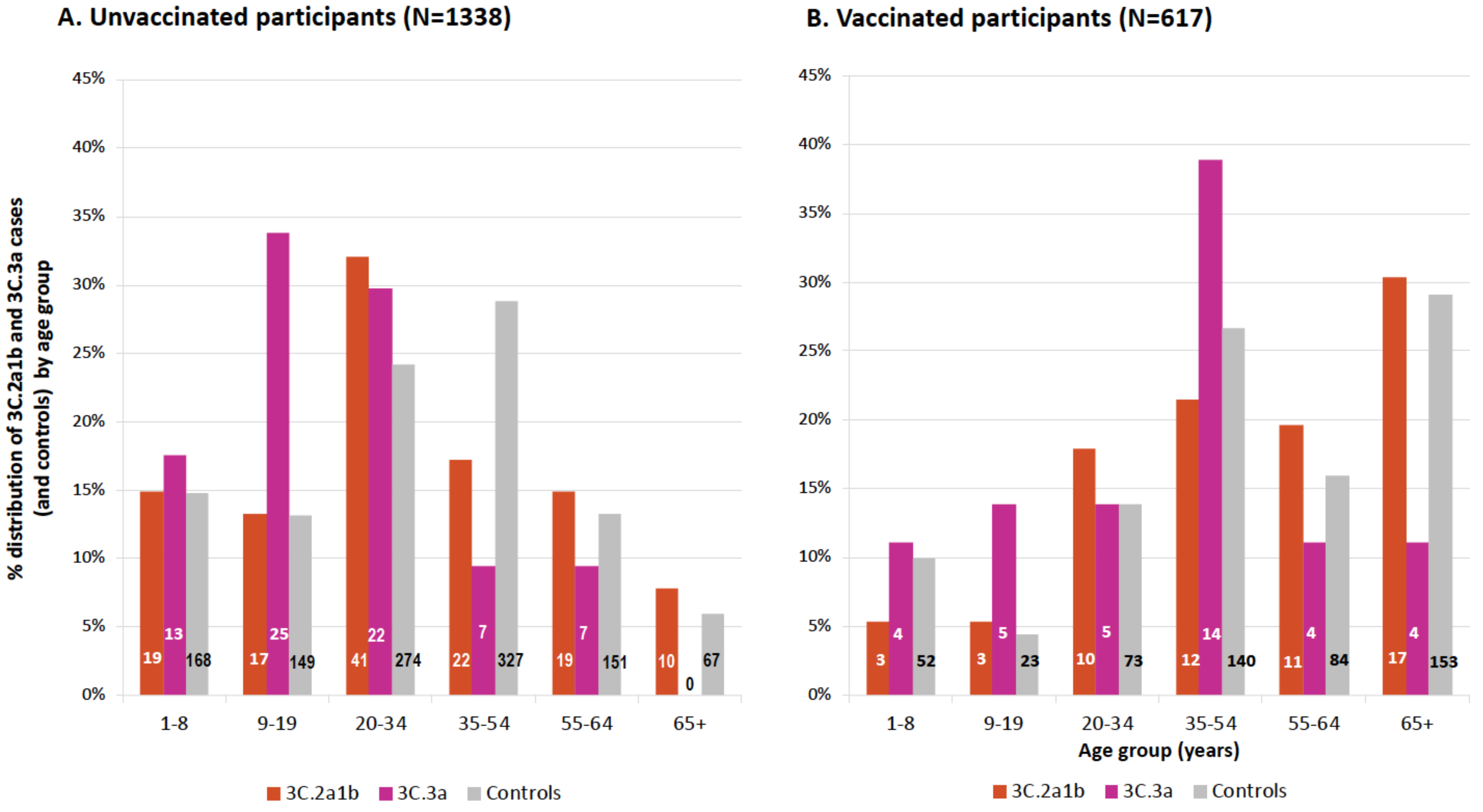

The percentage distribution of clade 3C.2a1b and clade 3C.3a cases (and influenza test-negative controls) by age subset, with stratification for those considered unvaccinated (A) or vaccinated (B). Vaccination status is defined by self-report of at least one dose of influenza vaccine ≥2 weeks prior to onset of influenza-like illness (ILI). Participants vaccinated <2 weeks before ILI onset are excluded.

Supplement Figure S15. Percentage vaccinated among clade 3C.2a1b and clade 3C.3a cases and controls by age subset

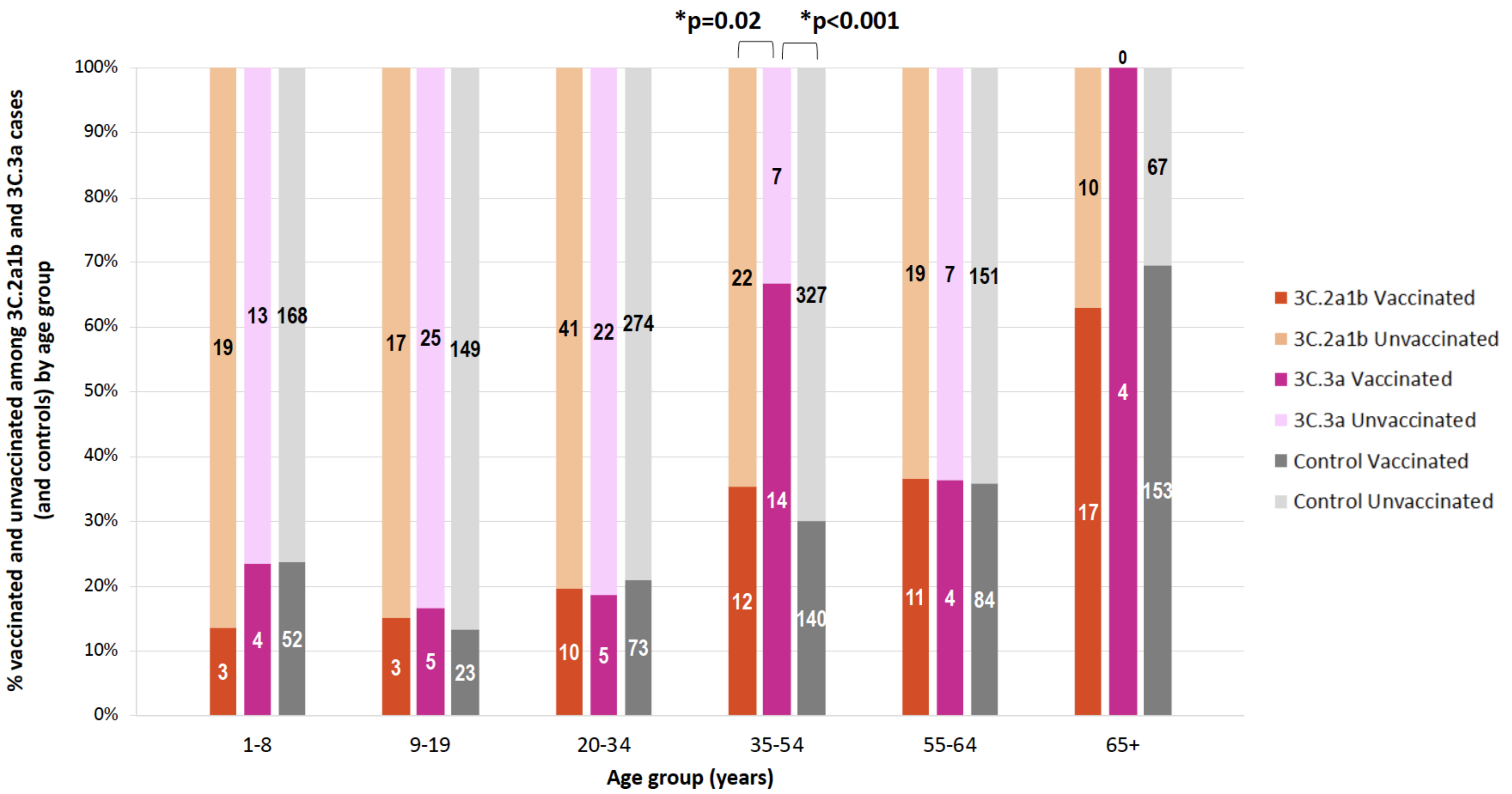

The percentage of contributing SPSN participants considered vaccinated among clade 3C.2a1b and clade 3C.3a cases and influenza test-negative controls, by age subset (N=1955). For each age subset, the proportion considered vaccinated vs. unvaccinated is compared between 3C.3a vs. 3C.2a1b cases and between each of these A(H3N2) genetic clades vs influenza test-negative controls. Vaccination status is defined by self-report of at least one dose of influenza vaccine ≥2 weeks prior to onset of influenza-like illness (ILI). Participants vaccinated <2 weeks before ILI onset are excluded. The number of participants within each stratum is overlaid on each column. Asterisks indicate comparisons achieving statistical significance with the corresponding p value displayed. No other comparisons of the odds of being vaccinated vs. unvaccinated between 3C.3a and 3C.2a1b cases or between 3C.3a or 3C.2a1b cases and controls were statistically significant at p<0.05.

Supplement Figure S16. Influenza A(H3N2) vaccine effectiveness by single year of age, 2016/17, 2017/18 and 2018/19

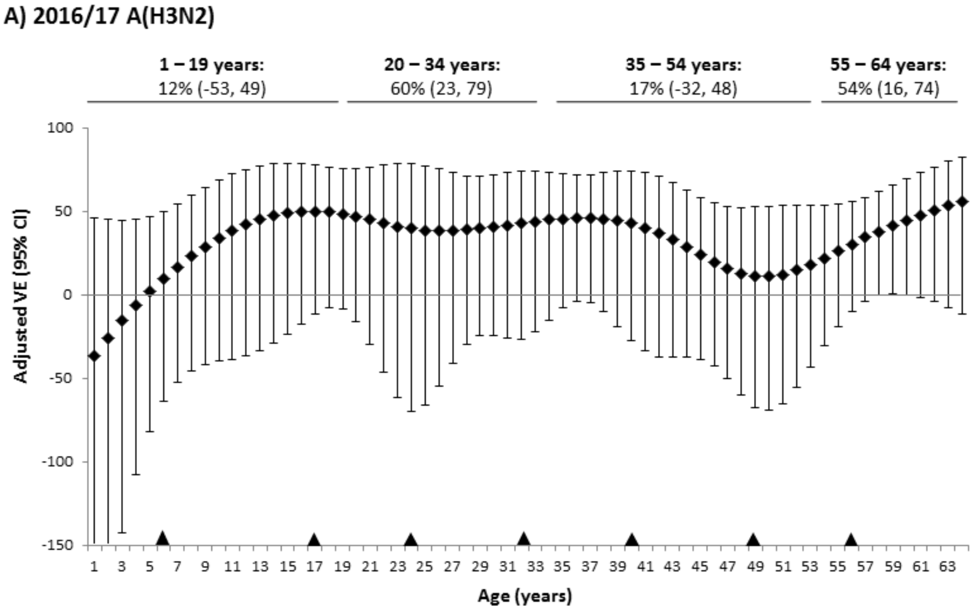

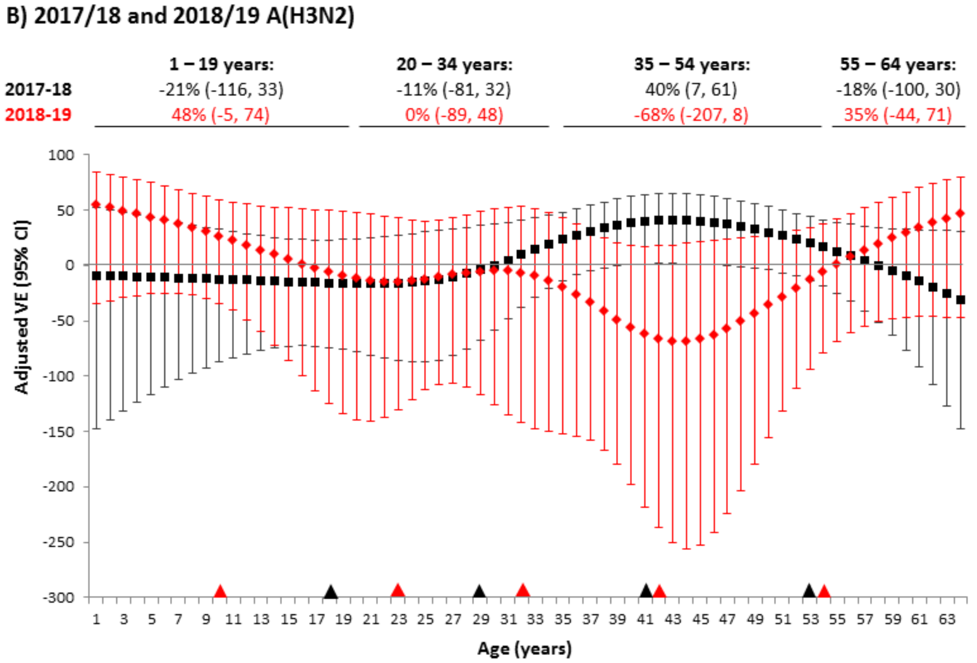

Adjusted vaccine effectiveness (VE) estimates with 95% confidence intervals by single year of age for A(H3N2) viruses in 2016/17 (panel A) and 2017/18, with mirror image findings for 2018/19 overlaid (panel B) are presented for Canadian Sentinel Practitioner Surveillance (SPSN) participants 1-64 years (for consistency with the display for 2018/19). Age is smoothed as a natural cubic spline function with knots at equal percentiles of age, demarcated by triangles on the x axis (red for 2018/19). Note that A(H3N2) viruses in 2016/17 were comprised of ~75% mixed genetic variants of clade 3C.2a1 and in 2017/18 of ~85% clade 3C.2a2 (both Y159 shielded by adjacent 160T glycosylation and F193). Note that the egg-adapted 3C.2a vaccine used in both 2016/17 and 2017/18 was also Y159 shielded and F193. Methods are detailed in Supplement S2. Adjusted VE estimates by age subsets as re-defined for A(H3N2) viruses in 2018/19 are also overlaid for comparison. Note also that age is at the time of specimen collection in the specified season, without reference or correction for different corresponding birth years by season. Except where otherwise specified, all VE estimates are adjusted for province, specimen collection interval, and calendar time and additionally for age as 1-8 and 9-19 years for the subset 1-19 years.

Supplement S17. References

1. Shu Y, McCauley J. GISAID: Global initiative on sharing all influenza data – from vision to reality. Euro Surveill. 2017;22(13).

2. Statistics Canada. Table 17-10-0005-01: Population estimates on July 1st, by age and sex. Ottawa: Statistics Canada. [Accessed: 29 October 2019]. Available from: https://www150.statcan.gc.ca/t1/tbl1/en/tv.action?pid=1710000501

3. Skowronski DM, Leir S, Sabaiduc S, Murti M, Dickinson JA, Olsha R, et al. Interim estimates of 2018/19 vaccine effectiveness against influenza A(H1N1)pdm09, Canada, January 2019. Euro Surveill. 2019;24(4).

4. Canadian Sentinel Practitioner Surveillance Network (SPSN). Influenza vaccine effectiveness estimates. Vancouver: BC Centre for Disease Control. [Accessed: 29 October 2019]. Available from: http://www.bccdc.ca/health-info/diseases-conditions/influenza/sentinel-network-spsn

5. Firth D. Bias reduction of maximum likelihood estimates. Biometrika 1993;80:27-38.

6. Heinze G, Schemper M. A solution to the problem of separation in logistic regression. Statist Med 2002;21:2409-2419.

7. Devika S, Jeyaseelan L, Sebastian G. Analysis of sparse data in logistic regression in medical research A newer approach. Journal of postgraduate medicine. 2016;62(1):26 10.4103/0022-3859.173193.

8. Chen Y, Cui D, Zheng S, Yang S, Tong J, Yang D, et al. Simultaneous detection of influenza A, influenza B, and respiratory syncytial viruses and subtyping of influenza A H3N2 virus and H1N1 (2009) virus by multiplex real-time PCR. J Clin Microbiol. 2011;49(4):1653-6.

9. Real-Time RT-PCR (rRT-PCR) protocol for influenza, CDC; PS-025, R-1D Effective 11 June 2012

10. Pabbaraju K, Wong S, Wong AA, Appleyard GD, Chui L, Pang XL, et al. Design and validation of real-time reverse transcription-PCR assays for detection of pandemic (H1N1) 2009 virus. J Clin Microbiol. 2009;47(11):3454-60.

11. Ndifon W, Wingreen NS, Levin SA. Differential neutralization efficiency of hemagglutinin epitopes, antibody interference, and the design of influenza vaccines. Proc Natl Acad Sci. USA. 2009;106(21):8701-6.

12. Popova L, Smith K, West AH, Wilson PC, James JA, Thompson LF, et al. Immunodominance of antigenic site B over site A of hemagglutinin of recent H3N2 influenza viruses. PLoS One. 2012;7(7):e41895.

13. Koel BF, Burke DF, Bestebroer TM, van der Vliet S, Zondag GC, Vervaet G, et al. Substitutions near the receptor binding site determine major antigenic change during influenza virus evolution. Science. 2013;342(6161):976-9.

14. An Y, McCullers JA, Alymova I, Parsons LM, Cipollo JF. Glycosylation analysis of engineered H3N2 influenza A virus hemagglutinins with sequentially added historically relevant glycosylation sites. J Proteome Res. 2015;14(9):3957-69.

15. Tate MD, Job ER, Deng YM, Gunalan V, Maurer-Stroh S, Reading PC. Playing hide and seek: how glycosylation of the influenza virus hemagglutinin can modulate the immune response to infection. Viruses. 2014;6(3):1294-316.

16. Abe Y, Takashita E, Sugawara K, Matsuzaki Y, Muraki Y, Hongo S. Effect of the addition of oligosaccharides on the biological activities and antigenicity of influenza A/H3N2 virus hemagglutinin. J Virol. 2004;78(18):9605-11.

17. Price MN, Dehal PS, Arkin AP. FastTree 2--approximately maximum-likelihood trees for large alignments. PLoS One. 2010;5(3):e9490.

18. Rambaut A. FigTree v1.4.0, a graphical viewer of phylogenetic trees. Edinburgh: University of Edinburgh. [Accessed: 29 October 2019]. Available from: http://tree.bio.ed.ac.uk/software/figtree/

19. Nextstrain. Real-time tracking of influenza A/H3N2 evolution. [Accessed: 29 October 2019]. Available from: https://nextstrain.org/flu/seasonal/h3n2/ha/6y

20. Worldwide Influenza Centre, Francis Crick Institute. Annual and interim reports. London: Francis Crick Institute. [Accessed: 29 October 2019]. Available from: https://www.crick.ac.uk/research/worldwide-influenza-centre/annual-and-interim-reports/

21. European Centre for Disease Prevention and Control (ECDC). Influenza virus characterization, summary Europe, June 2019. Stockholm: ECDC. [Accessed: 29 October 2019]. Available from: https://www.ecdc.europa.eu/en/publications-data/influenza-virus-characterisation-summary-europe-june-2019

22. World Health Organization. WHO recommendations on the composition of influenza virus vaccines Geneva: WHO. [Accessed: 29 October 2019]. Available from: http://www.who.int/influenza/vaccines/virus/recommendations/en/

23. Katoh K, Standley DM. MAFFT multiple sequence alignment software version 7: improvements in performance and usability. Mol Biol Evol. 2013;30(4):772-80.
